# Cellular Oxidative Stress Response Controls the Antiviral and Apoptotic Programs in Dengue Virus-Infected Dendritic Cells

**DOI:** 10.1371/journal.ppat.1004566

**Published:** 2014-12-18

**Authors:** David Olagnier, Suraj Peri, Courtney Steel, Nadine van Montfoort, Cindy Chiang, Vladimir Beljanski, Michael Slifker, Zhong He, Carmen N. Nichols, Rongtuan Lin, Siddharth Balachandran, John Hiscott

**Affiliations:** 1 Vaccine & Gene Therapy Institute of Florida, Port St. Lucie, Florida, United States of America; 2 Fox Chase Cancer Center, Philadelphia, Pennsylvania, United States of America; 3 Lady Davis Institute, Jewish General Hospital, McGill University, Montreal, Quebec, Canada; The University of Chicago, United States of America

## Abstract

Dengue virus (DENV) is a re-emerging arthropod borne flavivirus that infects more than 300 million people worldwide, leading to 50,000 deaths annually. Because dendritic cells (DC) in the skin and blood are the first target cells for DENV, we sought to investigate the early molecular events involved in the host response to the virus in primary human monocyte-derived dendritic cells (Mo-DC). Using a genome-wide transcriptome analysis of DENV2-infected human Mo-DC, three major responses were identified within hours of infection - the activation of IRF3/7/STAT1 and NF-κB-driven antiviral and inflammatory networks, as well as the stimulation of an oxidative stress response that included the stimulation of an Nrf2-dependent antioxidant gene transcriptional program. DENV2 infection resulted in the intracellular accumulation of reactive oxygen species (ROS) that was dependent on NADPH-oxidase (NOX). A decrease in ROS levels through chemical or genetic inhibition of the NOX-complex dampened the innate immune responses to DENV infection and facilitated DENV replication; ROS were also essential in driving mitochondrial apoptosis in infected Mo-DC. In addition to stimulating innate immune responses to DENV, increased ROS led to the activation of bystander Mo-DC which up-regulated maturation/activation markers and were less susceptible to viral replication. We have identified a critical role for the transcription factor Nrf2 in limiting both antiviral and cell death responses to the virus by feedback modulation of oxidative stress. Silencing of Nrf2 by RNA interference increased DENV-associated immune and apoptotic responses. Taken together, these data demonstrate that the level of oxidative stress is critical to the control of both antiviral and apoptotic programs in DENV-infected human Mo-DC and highlight the importance of redox homeostasis in the outcome of DENV infection.

## Author Summary

Dengue virus (DENV), the leading arthropod-borne viral infection in the world, represents a major human health concern with a global at risk population of over 3 billion people. Currently, there are no antivirals or vaccines available to treat patients with dengue fever, nor is it possible to predict which patients will progress to life-threatening severe dengue fever. Markers associated with oxidative stress responses have been reported in patients with severe DENV infection, suggesting a relationship between oxidative stress and viral pathogenesis. In order to uncover biological processes that determine the outcome of disease in patients, we utilized human dendritic cells, the primary target of DENV infection, in an *in vitro* model. Transcriptional analysis of pathways activated upon *de novo* DENV infection revealed a major role for cellular oxidative stress in the induction of antiviral, inflammatory, and cell death responses. We also demonstrated that antioxidant mechanisms play a critical role in controlling antiviral and cell death responses to the virus, acting as feedback regulators of the oxidative stress response. This report highlights the importance of oxidative stress responses in the outcome of DENV infection, and identifies this pathway as a potential new entry-point for treating dengue-associated diseases.

## Introduction

Dengue virus (DENV) is the leading arthropod-borne viral infection in the world, and represents a major global human health concern. DENV is endemic in more than 100 countries with up to 3 billion people in tropical regions of the world at risk of infection [1]–[3]. Recently, DENV has expanded its global range, with long-term outbreaks in South America and reintroduction into North America through Florida and Texas, with each of these outbreaks accompanied by increased disease severity. Of the estimated 50–100 million annual cases, the majority of infected individuals develop a self-limiting febrile illness, but approximately 500,000 clinical cases result in more severe manifestations, such as DENV-induced hemorrhagic fever and shock syndrome [1], leading to 25–50,000 deaths per year [4]. The pathogenesis of dengue is incompletely understood and the factors that determine whether infection manifests as self-limiting dengue fever or progresses to life-threatening illness remains unanswered.

Dengue is an RNA virus of the *Flaviviridae* family with 4 closely related serotypes that exhibit inter- and intra-serotypic genetic diversity [5]–[9]. Innate recognition of DENV involves a spectrum of pattern recognition receptors (PRR) that sense conserved molecular components termed pathogen associated molecular patterns (PAMP), and together orchestrate antiviral responses to the viral infection. The cytoplasmic helicases RIG-I and MDA-5 have a central role in the host response to DENV by contributing to DENV protection in hepatocytes [10]. Additionally, TLR3 and TLR7 recognize DENV RNA and mount a rapid protective immune response in human monocytic cells and plasmacytoid dendritic cells, respectively [11], [12]. Signaling through these different cellular sensors leads to the activation of the interferon pathway that restricts viral proliferation and contributes to the establishment of adaptive immune responses *via* NF-κB-mediated cytokine and chemokine release [13]–[16]. Interestingly, the host immune response, activated in response to DENV infection, not only mediates protection against disease, but also contributes to disease severity [1]. For example, high levels of circulating pro-inflammatory cytokines such as IL-1β or TNF-α in DENV-infected patients correlates with severe dengue fever, compared to patients suffering with mild dengue fever [17].

Reactive oxygen species (ROS) production, generated as a consequence of microbial invasion, has long been known to exert an antimicrobial effect in phagocytes [18]. The activation of the antiviral and inflammatory signaling pathways has also been linked with the production of ROS [19]–[23], which include oxygen ions and peroxides that are produced as byproducts of aerobic metabolism. Because of the high chemical reactivity of ROS, cells possess scavenger antioxidant mechanisms that maintain redox homeostasis [24]–[26]. Signaling pathways downstream of ROS detection activate the transcription factor nuclear factor-erythroid 2-related factor 2 (Nrf2) [24]–[26], which binds antioxidant response elements (ARE) within the promoters of genes encoding antioxidant and detoxifying enzymes. Nrf2-dependent antioxidant genes act synergistically to reduce oxidative stress by quenching ROS [24]–[26].

Increased generation of ROS and changes in redox homeostasis have been described in the context of many viral infections [23], [27]–[33] and the failure to maintain an appropriate redox balance contributes to viral pathogenesis through alterations of biological structures and the massive induction of cell death [34]–[36]. In the flavivirus family, hepatitis C virus (HCV) has been shown to promote oxidative stress and manipulate antioxidant systems, leading to chronic disease [31], [37], [38]. As well, DENV was shown to stimulate oxidative stress in hepatocytes leading to production of the chemokine CCL5 and to activation of the transcriptional regulator C/EBP beta [39]. Furthermore, HepG2 xenografted SCID mice presented alterations in oxidative stress status and increased inflammatory cytokines following DENV infection [40]. More recently, oxidative stress-induced damage and alterations in redox status have been associated with increased disease severity in DENV-infected patients, suggesting a possible role for oxidative stress in DENV-induced pathogenesis [41]–[44]. Interestingly, circulating monocytes from glucose-6-phosphate dehydrogenase (G6PD)-deficient patients, displayed an increased susceptibility to DENV infection and replication [45]. The G6PD deletion affects ROS production, thus linking cellular oxidative state and susceptibility to DENV infection. Altogether, these observations underline the importance of the redox homeostasis in DENV infection and suggest an important interplay between the generation of oxidative stress and the immunopathology of dengue disease.

Initial contact between DENV and innate immune cells plays an essential role in the outcome of the infection. Indeed, DENV infection pushes monocytes towards a CD16+ inflammatory phenotype that facilitates plasmablast differentiation and induction of anti-DENV antibody responses [46]. Given the importance of DC in bridging the innate and adaptive immune response, and since DC in the skin and peripheral blood are the first target cells for DENV after transmission *via* a mosquito bite [47]–[49], evaluation of the early molecular events in DC is crucial to the understanding of DENV pathogenesis. In the present study, we generated in-depth transcriptome analysis, coupled with biochemical and functional analyses of the early host response to DENV infection in primary Mo-DC. DENV infection triggered an NADPH-oxidase (NOX)-dependent oxidative stress response that was required for the activation of IRF3/7/STAT1 and NF-κB-mediated antiviral responses and for mitochondrial-dependent apoptosis. Furthermore, we have identified a critical role for the transcription factor Nrf2 in regulating both antiviral and inflammatory gene response to the virus by feedback modulation of oxidative stress. Overall, these studies highlight the importance of redox homeostasis in the outcome of DENV infection.

## Results

### DENV2 highly infects Mo-DC and generates a broad antiviral response

**Figure 1:**
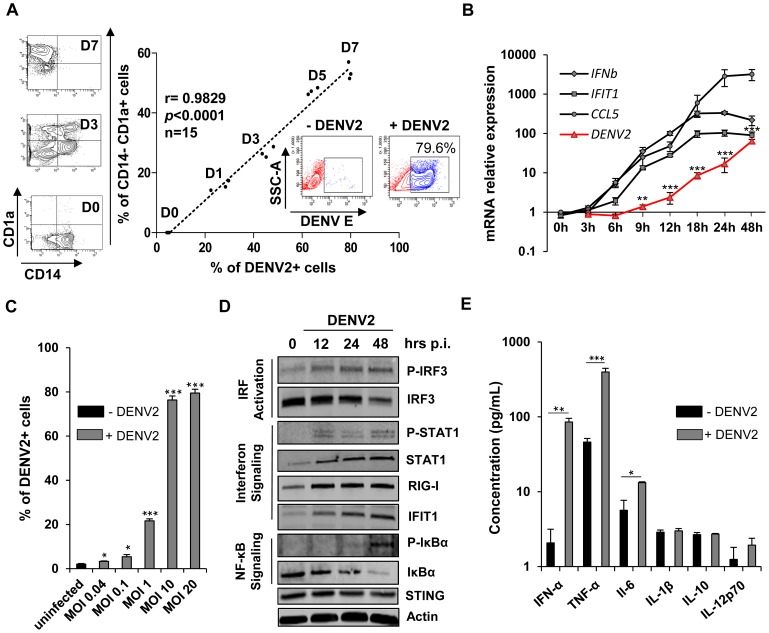
Mo-DC are highly susceptible to DENV infection and generate a broad antiviral and inflammatory response. (A) The correlation between the percentage of infected cells determined by intracellular staining of DENV E protein expression and the percentage of CD14^−^ CD1a^+^ Mo-DC was calculated during the course of Mo-DC differentiation (n = 15; Spearman test). The FACS panels on the left hand side represent the progression of Mo-DC differentiation at days 0, 3 and 7. (B) DENV RNA levels as well as *IFN-β*, *IFIT1*, and *CCL5* gene expression levels were determined by qPCR at various times of DENV2 infection (MOI 10). P values were determined based on the comparison with cells at time 3 h after infection. Data are from one experiment performed on three individual donors. (C) Mo-DC were infected with increasing amounts of virus (MOI 0.04-MOI 20) for 24 h. Percentage of DENV2-infected cells was determined by intracellular staining (ICS) of DENV E protein expression using flow cytometry. Data are the means ± SEM from one experiment performed on three different donors. (D) Mo-DC were challenged with DENV2 (MOI 20) and antiviral and inflammatory responses were examined by immunoblotting. Data are from one representative experiment. (E) Cytokine release was evaluated by cytokine bead array (CBA) 24 h after DENV2 challenge (MOI 10) in the supernatants of infected cells. P values were determined based on the comparison with uninfected cells. Data are the means ± SEM from one experiment performed on three different donors.

An *in vitro* model of *de novo* DENV infection was established using primary human monocytes differentiated *in vitro* with Mo-DC-differentiation medium containing GM-CSF and IL-4. Primary CD14^+^ CD1a^−^ monocytes were less permissive to DENV2 infection, whereas infectivity increased progressively as the cells differentiated toward the Mo-DC (CD14^−^ CD1a^+^) phenotype (4.66±0.45% of DENV+ cells in monocytes at day 0 *vs* 79.6±0.47% in Mo-DC at day 7) (Fig. 1A). A strong statistical correlation between a CD14^−^CD1a^+^ phenotype and DENV infection was confirmed by the nonparametric Spearman test (r = 0.9829; *p*<0.0001; n = 15). DENV2 viral RNA accumulation was detected after a lag period of 6 h and increased exponentially thereafter (Fig. 1B), which corroborates a previous report demonstrating release of infectious particles [50]. Prior to the onset of detectable DENV replication, an antiviral response was mounted by the infected Mo-DC population, as demonstrated by the increase in *IFN-β*, *IFIT1* and *CCL5* gene expression (Fig. 1B). DENV infected Mo-DC in a dose dependent manner to a maximum of ∼80% infectivity at a MOI of 20 (Fig. 1C). As a consequence of early virus sensing, a broad antiviral and inflammatory response was generated as shown by the phosphorylation of IRF3 and STAT1 (Fig. 1D) and significant release of IFN-α, TNF-α and IL-6 (Fig. 1E) by the infected cells. Previous studies reported cleavage of the endoplasmic reticulum adaptor STING upon DENV infection in Mo-DC [51]. However, in our experimental model and with the viral strain used, a modest 20% decrease in STING expression was observed at 48 h after infection (Fig. 1D). Altogether these data demonstrate that DENV-infected Mo-DC generate a broad host response and secrete an array of antiviral and inflammatory cytokines in response to the virus.

### Transcriptome kinetics of the host response to DENV2 infection

**Figure 2:**
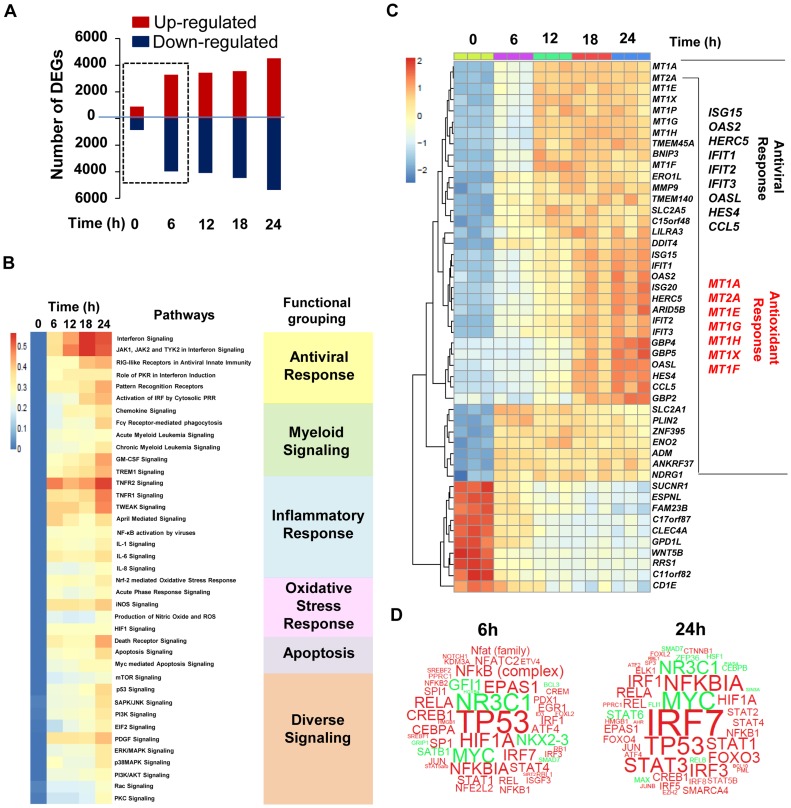
Transcriptome analysis of the host response to DENV2 infection in Mo-DC. Mo-DC were infected with DENV2 (MOI 20) for designated periods of time. Samples were analyzed by Illumina gene expression array and differentially expressed genes (DEGs) that satisfied a p value (<0.05) with ≥1.3 fold change (up or down) were selected. (A) Waterfall plot representing the total number of up-regulated and down-regulated genes at each time point. (B) Heat map shows statistically significant canonical pathways (Ingenuity Pathway Analysis Software) commonly regulated at 6 h, 12 h, 18 h and 24 h when compared to baseline. Genes that had adjusted p-value <0.05 at each time point and fold change >1.3 or <−1.3 and associated with a canonical pathway in Ingenuity's Knowledge Base were used for pathway analysis. Heat map colors represent the ratio of regulated genes/pathway genes after dengue infection (red and blue correspond to over- and under-represented, respectively). The over-representation test was performed using Fisher Exact Test. Statistical significance achieved at p<0.05. The data are representative of one experiment performed on three different donors. (C) Gene expression heatmap of the top 50 differentially expressed genes induced by dengue infection in Mo-DC at various times when compared to baseline. Genes are selected as differentially expressed in at least one comparison following ANOVA F test as implemented in the LIMMA package. The scale shows the level of gene expression where red and blue correspond to up- and down-regulation respectively. A panel of antiviral (black) and antioxidant (red) DEG is represented on the right hand side of the heatmap. (D) Word clouds representing potentially activated (red)/inhibited (green) transcription factors at 6 h and 24 h after DENV2 challenge. IPA Upstream Regulator Analysis was used to identify molecules upstream of the genes in the data set that could explain the detected expression changes. The p-value of overlap, which measures the enrichment of network-regulated genes in the data set, is represented by the size of the word. The activation z-score which predicts likely regulating molecules was used to color the predicted activation state.

To characterize signaling pathways involved in the host intrinsic response to DENV2 infection, a transcriptome analysis of DENV-infected Mo-DC was performed; Fig. 2A represents a waterfall plot of differentially expressed genes (DEG; selected based on fold change >±1.3, *p* value <0.05) after DENV2 infection. Most changes in gene expression appeared early, with over 7000 genes either up- or down-regulated by 6 h after infection (Fig. 2A). Pathway analysis identified multiple canonical networks coordinately regulated at all times after infection; the expected IFN/IRF antiviral pathways as well as the NF-κB-dependent pro-inflammatory pathways were all highly enriched after *de novo* DENV2 infection (Fig. 2B). We also noticed an enrichment of networks associated with the generation of a pro- and anti-oxidant stress response (Fig. 2B). Further gene analysis represents the top 50 DEG over time following DENV infection (Fig. 2C); among the top up-regulated genes, two subclasses predominated – interferon-stimulated genes (ISGs) such as *ISG15*, *IFIT1*, *IFIT2*, *IFIT3*, *OASL*, *OAS2*, *CCL5*, *HES4* (presented in black) and more surprisingly a large set of antioxidant genes belonging to the metallothionein family including *MT1A*, *MT2A*, *MT1E*, *MT1X*, *MT1G*, *MT1H*, and *MT1F* (presented in red) (Fig. 2C). Based on the regulation of gene networks activated or repressed after DENV2 infection, Fig. 2D illustrates a word cloud map of possibly activated (red) or inhibited (green) transcription factors controlling gene networks at 6 h and 24 h after DENV2 challenge (Fig. 2D). At 6 h after infection, two subclasses of transcription factors predominated: 1) transcription factors associated with cellular stress-responses including TP53 (p53), EPAS1, HIF1A and NFE2L2 (Nrf2); and 2) transcriptional regulators associated with the antiviral program including IRF1/3/7, STAT1/ISGF3 and NF-κB complex (Fig. 2D). By 24 h post-infection, the activity of stress-related transcription factors decreased, with the exception of TP53, while transcription factors driving the antiviral response - predominantly IRF7 and NF-κB - were highly active (Fig. 2D).

**Figure 3:**
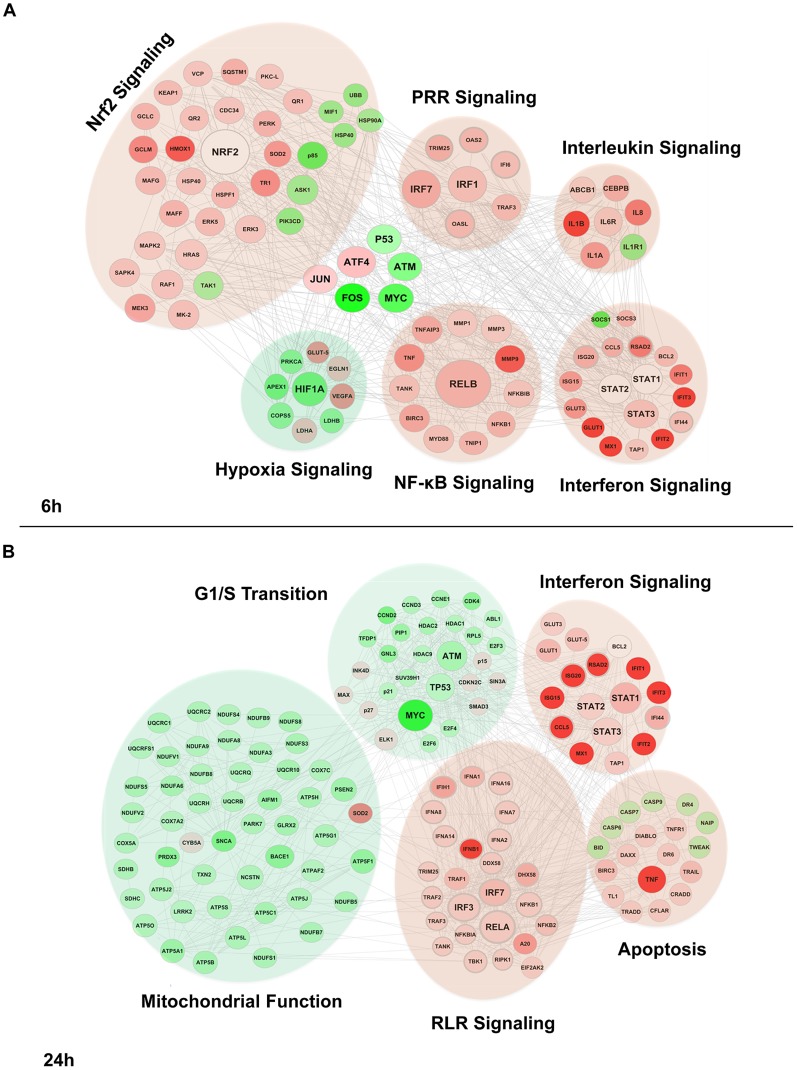
Functional characterization of genes differentially expressed by DENV2 infection. Differentially expressed genes were subjected to Ingenuity Pathway Analysis for both 6 h (A) and 24 h (B) time points based on *p*-values (<0.001) and fold change ≥±1 (log2). Red represent genes induced by DENV, and green indicated genes downregulated by the virus; the intensity of the color is representative of the fold change. Larger circles indicate transcription factors. Genes enriched for a pathway are represented as a cloud.

A Fluidigm BioMark high throughput qPCR assay encompassing a cross-section of genes identified in the genomic analysis (S1 Table) was used to validate the transcriptome data; the pattern of gene expression at various times after DENV2 infection was similar for three different donors (S1A Figure). Computational analysis identified different kinetics of IFN induction, as well as sustained up-regulation of chemokines, Th1 cytokines, ISGs and antiviral transcription factors (S1B Figure). A strong statistical correlation between the log fold change for the microarray values and the log fold change for the BioMark values was confirmed by a Spearman correlation test (S1C Figure) (r = 0.8399194; *p* = 4.576e-14; n = 49). In order to gain systems-wide insight into DENV-modulated transcriptome, a functional clustering (node analysis) (Fig. 3), as well as gene-pathway checkerboard analysis (S2 Figure) of DENV-induced DEGs was performed. This functional clustering identified at 6 h (Fig. 3A and S2A Figure) and 24 h (Fig. 3B and S2B Figure) a variety of transcriptional sub-networks and biological processes regulated by DENV. The Nrf2-mediated oxidative stress response pathway, the top differentially regulated pathway in DENV-infected Mo-DC at 6 h (S2A Figure), was triggered prior to the onset of viral replication and intersected with other pathways such as NF-κB, IRF and STAT signaling (Fig. 3A and S2A Figure). At the same time, hypoxia pathway controlled by the transcription factor HIF1-α was predominantly down regulated (Fig. 3A and S2A Figure). By 24 h the activity of the Nrf2-driven pathway decreased, whereas the expansion and increased interaction among the antiviral, inflammatory and death response networks predominated (Fig. 3B and S2B Figure). Concomitantly, genes related to mitochondrial function were all significantly down regulated and presumably associated with an increase in the apoptotic response (Fig. 3B and S2B Figure).

### DENV-induced NOX-derived ROS accumulation is essential for induction of innate immune responses

**Figure 4:**
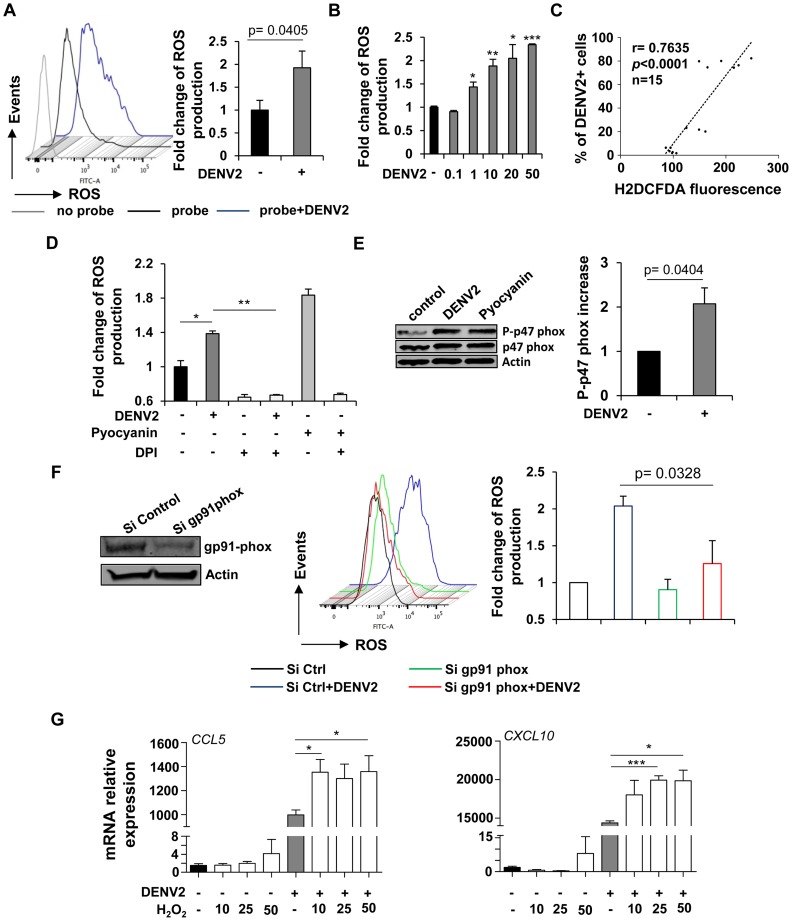
DENV-infected cells accumulate intracellular NOX-derived ROS which potentiate the immune response. (A) ROS generation in DENV-infected Mo-DC (MOI 20) was monitored by FACS using CM-H2DCFDA (1 µM) 18 h after infection. Data represent the mean ± SEM from experiments performed on four different donors. (B) Accumulation of ROS was measured by flow cytometry using CM-H2DCFDA (1 µM) 24 h after infection with increasing amount of virus. Data represent the mean ± SEM from one experiments performed on three different donors. P values were determined based on the comparison with uninfected cells (C) The correlation between the percentage of infected cells determined by intracellular staining of DENV E protein expression and the intracellular accumulation of ROS measured by DCFDA staining was calculated (n = 15; Spearman test). (D) Oxidative stress generation was detected by flow cytometry using the CM-H2DCFDA (1 µM) fluorescent probe on Mo-DC pretreated with DPI (3 µM) and infected with DENV2 (MOI 20) for 3 h or treated with the stress-inducer Pyocyanin (100 µM). Data represent the geometric mean fluorescence ± SEM from an experiment performed on two different donors. (E) Expression level of the phosphorylated NADPH-oxidase p47 subunit was assessed by immunoblotting 3 h after DENV2 infection. Histograms represent the fold change ratio of phosphorylated-p47 over total p47. Data are the means ± SEM of independent experiments performed on three different donors. P value was determined based on the comparison with uninfected cells. (F) Mo-DC were transfected with control or gp91 phox siRNA and 48 h later were infected with DENV2 (MOI 20) for 18 h. ROS accumulation was measured by FACS using the CM-H2DCFDA fluorescent probe. Data are the means ± SEM of three independent experiments performed on different donors. P value was determined based on the comparison with cells infected with DENV and transfected with the control siRNA sequences (G) Mo-DC were treated with increasing concentrations of hydrogen peroxide (10–50 µM) in the presence or absence of DENV2 (MOI 20). CCL5 and CXCL10 mRNA expression levels were monitored by qPCR 24 h following treatment and infection. Data represent the means ± SEM from one experiment performed on three individual donors. Experiment has been repeated twice. P values were determined based on the comparison with DENV2-infected cells.

The role of reactive oxygen species (ROS) as specific second messengers in signaling cascades involved in cell proliferation, differentiation and immune activation has been well documented [52]. In light of the array data and to evaluate if ROS are involved in the recognition of DENV, Mo-DC were infected and ROS formation was monitored by flow cytometry using the oxidant-sensitive fluorescent detection probe CM-H2DCFDA. ROS production was induced in DENV-infected Mo-DC, as reflected in the 2-fold increase in DCF fluorescence detected by FACS at 18 h after infection (p = 0.0405) (Fig. 4A). Also, DENV infection increased intracellular ROS accumulation in a dose dependent manner (Fig. 4B). A strong statistical correlation between DENV infection and the accumulation of ROS was confirmed by the nonparametric Spearman test (r = 0.7635; p<0.0001; n = 15) (Fig. 4C). Although ROS are generated intracellularly, the primary sources of ROS are plasma membrane oxidases, particularly NADPH oxidases. ROS were detected as early as 3 h after infection, and ROS production was suppressed by pre-treatment with the antioxidant diphenyleneidonium chloride (DPI), an NADPH-oxidase (NOX) inhibitor (Fig. 4D). ROS production was independently confirmed in Mo-DC by the use of pyocyanin (N-methyl-1-hydroxyphenazine), an oxidative stress inducer, as denoted by the 1.8 fold increase in ROS generation at 3 h after stimulation (Fig. 4D). The involvement of NOX in DENV-induced ROS accumulation was further confirmed by the increased phosphorylation of the p47 subunit of the NADPH-oxidase (p = 0.0404) (Fig. 4E). Interference with NADPH-oxidase activity using siRNA-mediated silencing of the catalytic gp91phox subunit limited ROS accumulation in response to *de novo* DENV infection (p = 0.0328) (Fig. 4F). To examine whether cellular oxidative stress impacted the immediate host response to DENV, we evaluated the effect of exogenous ROS addition on expression of DENV-induced antiviral genes. Treatment with increasing concentrations of hydrogen peroxide (H_2_O_2_) did not stimulate immune responses in Mo-DC; however addition of H_2_O_2_ moderately potentiated the elevation of DENV-induced antiviral gene expression (Fig. 4G).

**Figure 5:**
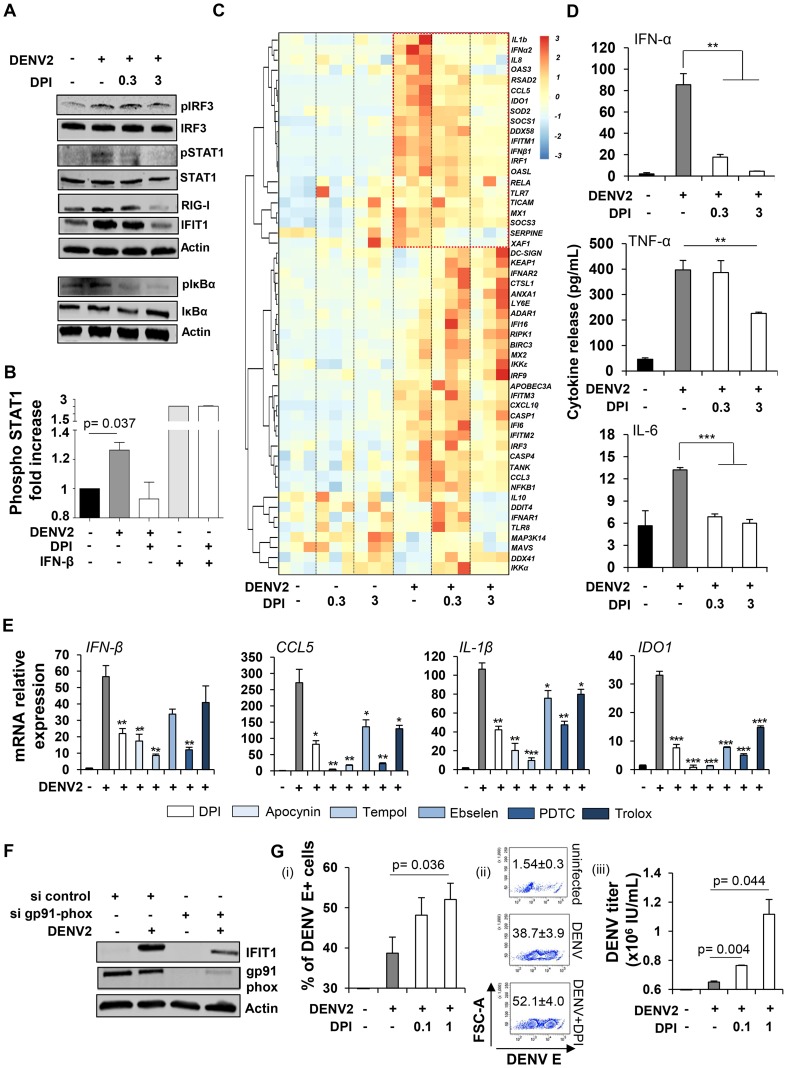
Cellular oxidative stress response is required to mediate DENV-induced innate immune responses. Mo-DC were pre-treated with DPI (0.3 or 3 µM) for 1 h, and subsequently infected with DENV2 (MOI 20) for 24 h. (A) Antiviral and inflammatory responses were monitored by immunoblotting. Data are representative of at least two independent experiments on separate donors. (B) Intracellular levels of phosphorylated STAT1 were detected by PhosFlow in Mo-DC infected for 24 h by DENV2 (MOI 20) or stimulated for 30 min with IFN-β (1000 IU/mL) and pre-treated or not with DPI (1 µM). Data represent the means ± SEM from two experiments performed on separate donors. (C) High throughput analysis of gene expression evaluated by qPCR BioMark analysis. Gene expression levels were calculated using the ΔΔCt method and gene-wise standardized expression (z-score) were generated for each gene. The scale represents z-score values where red shows an up-regulation and blue a down-regulation in gene expression. Data are representative of one experiment performed on three individual donors. Each box of the heatmap represents one donor. (D) Cytokine release was evaluated by cytokine bead array (CBA) on the supernatants of DENV infected cells pre-treated or not with DPI (0.3–3 µM). Data represent the means ± SEM from three individual donors. P values were determined based on the comparison with DENV2-infected cells. (E) Mo-DC were pre-treated with DPI (3 µM), Apocynin (3 mM), TEMPOL (3 mM), Ebselen (10 µM), PDTC (40 µM) and Trolox (5 µM) for 1 h, and subsequently infected with DENV2 (MOI 20) for 24 h. Antiviral and inflammatory gene expression was determined by qPCR. Data represent the means ± SEM from one experiment performed on three individual donors. P values were determined based on the comparison with DENV2-infected cells. (F) Mo-DC were transfected with control or gp91 phox siRNA and 48 h later were infected with DENV2 (MOI 20). IFIT1 and gp91 phox protein expression levels were measured by immunoblot analysis. Result is representative of one experiment. (G) Mo-DC were pre-treated with DPI (0.1–1 µM) for 1 h, and subsequently infected with DENV2 (MOI 1) for 48 h. Percentage of infected cells was determined by intracellular staining of DENV E protein (i–ii). DENV titers were determined by transferring supernatants from Mo-DC-infected cells on A549 cells and staining for DENV E protein (iii). DENV titers were expressed as the number of infectious units/mL. Data represent the means ± SEM of experiments performed on four (i–ii) and two different donors (iii).

Next, the role of ROS in triggering the early host response to DENV2 was evaluated by treating infected Mo-DC with increasing concentrations of DPI, an NADPH-oxidase inhibitor. Strikingly, phosphorylation of IRF3, STAT1 and IκBα, as well as the induction of ISGs such as RIG-I and IFIT1 – all markers of the antiviral response – were inhibited in a dose-dependent manner by DPI (Fig. 5A). The observation that NOX-inhibitor blocked DENV-induced immune response was further confirmed by quantitative intracellular measurement of STAT1 phosphorylation. Indeed, DPI prevented the increase in STAT1 phosphorylation detected by PhosFlow following DENV infection. Importantly, IFNβ-induced STAT1 phosphorylation was not affected by the DPI treatment (Fig. 5B). Using a customized BioMark chip, antiviral and inflammatory genes such as type I IFNs (*IFNA2*, *IFNB1*), pro-inflammatory cytokines and chemokines (*IL1β*, *CCL5*) and ISGs (*MX1, IFITM1/2/3, OASL, IDO1, OAS3, DDX58*) were inhibited by DPI in a dose dependent manner in DENV-infected cells (Fig. 5C, upper right box). Cytokine release (IFN-α, TNF-α and IL-6) was also impaired in the presence of the antioxidant molecule (Fig. 5D). The use of antioxidant molecules with different modes of action (S3A Figure) recapitulated the effect observed with DPI and impaired the induction of antiviral and inflammatory gene expression (Fig. 5E). Importantly, all antioxidant molecules tested in this panel did not affect cell survival, as shown in S3B Figure and S3C Figure. Inhibition of NADPH-oxidase activity using transient knock-down of the catalytic gp91phox subunit also decreased IFIT1 protein expression following *de novo* DENV infection (Fig. 5F). No increase in DENV RNA accumulation was detected in the presence of the NOX-inhibitor (3 µM) after 24 h of infection (S4 Figure). However, pre-treatment of cells with a higher concentration of DPI (30 µM) led to an increase in DENV viral RNA accumulation in the same conditions (S4 Figure). Importantly, DPI treatment resulted in increased DENV infectivity and replication at 48 h post-infection, as demonstrated by the increased number of DENV-infected cells (i–ii) and viral titers (iii) (Fig. 5G). The ROS-mediated induction of antiviral and inflammatory genes required live and replicating virus, since formalin-inactivation and UV-inactivation of DENV2 completely suppressed the induction of the immune response (S5A Figure and S5B Figure). Also, DPI inhibited antiviral and inflammatory responses induced by DENV2 strain 16681 (S5C Figure), indicating that ROS-mediated antiviral induction is a common feature of the DENV2 serotype and is not restricted to a specific strain. Collectively, these data demonstrate that DENV infection of Mo-DC triggers an intracellular accumulation of NOX-derived ROS, which are essential for the induction of the antiviral and inflammatory immune responses and the control of DENV infection.

### DENV-induced ROS drive mitochondrial apoptosis in Mo-DC and contribute to bystander cell maturation

**Figure 6:**
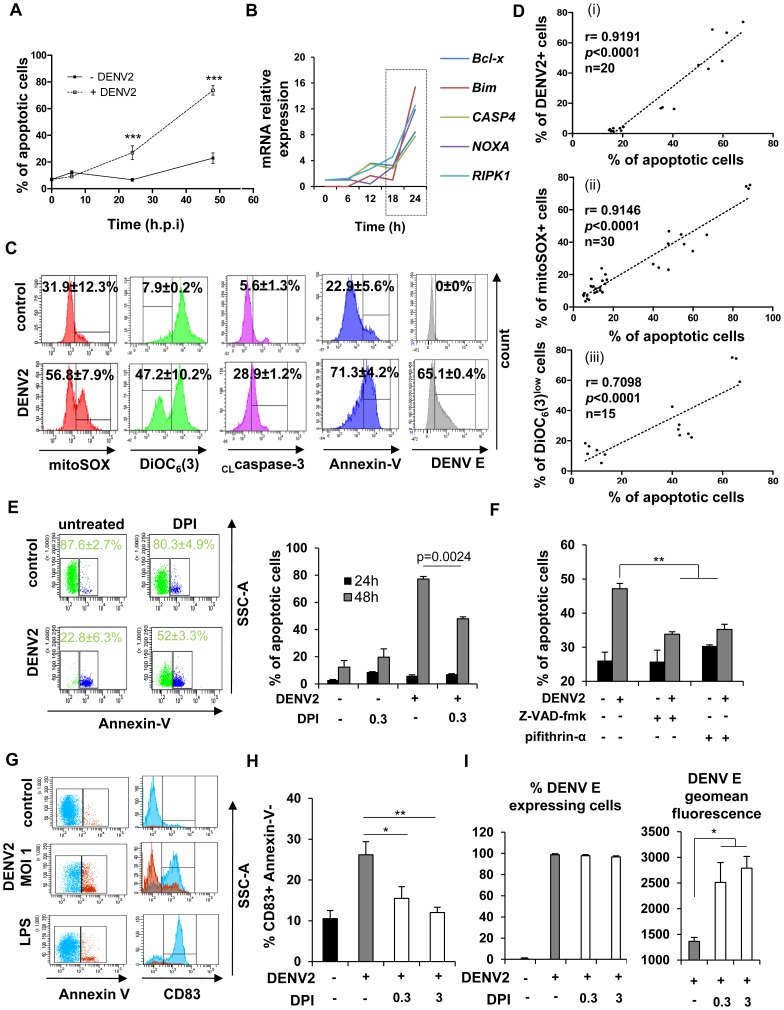
NOX-dependent ROS production triggers mitochondrial-dependent apoptosis in DENV-infected cells and activates bystander cells. (A) The percentage of apoptotic cells was assessed by Annexin-V staining at 6–48 h post-infection. Data are the means ± SEM from independent experiments performed in triplicate on five individual donors. P values were determined based on the comparison with uninfected cells at the appropriate time (B) mRNA relative expression of apoptosis-associated genes was detected by qPCR at various times post DENV2 infection. Values are representative of one donor. Experiment has been repeated on three individual donors. (C) Levels of mitoSOX red, DiOC_6_(3), cleaved caspase 3 (_CL_caspase3), Annexin-V and DENV E protein expression were evaluated by flow cytometry 48 h post-DENV infection. Values represent the means ± SEM from at least three individual donors. (D) The correlation between (i) the percentage of DENV2+ cells at 24 h and the percentage of apoptotic cells at 48 h; (ii) the percentage of apoptotic cells and the percentage of mitoSOX+ cells at 48 h, and (iii) the percentage of apoptotic cells and the percentage of DiOC_6_(3)^low^ cells at 48 h was calculated in Mo-DC using a Spearman test. (E) Percentage of apoptotic cells in DENV-infected Mo-DC was detected 48 h post-infection in the presence or absence of DPI (0.3 µM). Histograms represent the means ± SEM of three experiments performed in duplicate on three independent donors. (F) Percentage of apoptotic cells in DENV-infected Mo-DC was detected 48 h post-infection in the presence or absence of the p53 inhibitor pifithrin-α (10 µM) or the pan-caspase inhibitor Z-VAD-fmk (20 µM). Histograms represent the means ± SEM of one representative experiment performed in triplicate. P values were determined based on the comparison with DENV2-infected cells. (G) CD83 expression level was evaluated on Annexin-V^+^ (red) and Annexin-V^−^ (blue) DENV-infected cell population (MOI 1) (H) Mo-DC were pre-treated with increasing concentrations of DPI (0.3–3 µM) before DENV challenge (MOI 0.5). Percent of CD83^+^ Annexin-V^−^ cells was detected by flow cytometry 48 h after infection. P values were determined based on the comparison with DENV2-infected cells. Data are the means ± SEM of two experiments performed in triplicate. (I) Mo-DC pre-treated or not with increasing concentrations of DPI were cultured in the presence of DENV2 (MOI 20). After 24 h of infection, supernatants were collected and transferred for 8 h on naïve Mo-DC and cells were subsequently infected by DENV2 (MOI 20). DENV infection was assessed 24 h later by flow cytometry. The values are means ± SEM from one experiment performed in triplicate. P values were determined based on the comparison with DENV2 infected cells.

DENV-infected DC were clearly apoptotic, based on Annexin-V staining: 27±5.15% (infected) *vs* 6.69±1% (control) at 24 h and 73.65±4.2% (infected) *vs* 22.96±3.88% (control) at 48 h (Fig. 6A). Upregulation of mRNA levels for pro-apoptotic genes such as *BCLX*, *BIM*, and *CASP4* upon DENV infection (Fig. 6B) was consistent with the transcriptome analysis that identified the induction of apoptosis-associated pathways 24 h after DENV infection (Fig. 2B and Fig. 3B).

To assess the release of mitochondrial ROS, cells were stained with mitoSOX, a probe specific for mitochondria-derived ROS; the number of mitoSOX-positive cells increased from 31.9±12.3% (uninfected) to 56.8±7.9% (infected) at 48 h after infection. DiOC_6_ was also used to determine the loss of mitochondrial potential upon DENV infection: only 7.9±0.2% uninfected cells were positive, whereas 47.2±10.2% of infected cells were positive for mitochondrial depolarization. Consistent with the release of mitochondrial ROS and mitochondrial depolarization, intracellular levels of cleaved caspase-3 increased from 5.6±1.3% in uninfected cells to 28.9±1.2% in infected cells (Fig. 6C). Regression analysis indicated that the percentage of infected cells at 24 h correlated with the percentage of apoptotic cells at 48 h after infection (Fig. 6D (i)). Furthermore, both mitochondrial ROS release and mitochondrial depolarization were statistically associated with apoptosis induction (Fig. 6D (ii) and (iii)), thus demonstrating DENV-infected Mo-DC undergo mitochondrial-dependent apoptosis. When DC were pre-treated with the NOX-inhibitor DPI, a statistically significant decrease in apoptosis of DENV-infected cells was observed (∼80% for DENV only infection compared to ∼52% for DENV+DPI infection), indicating that mitochondrial-dependent apoptosis was also dependent, at least in part, on NOX-generated ROS (Fig. 6E). Based on the array data, a key sensor of cellular stress, the transcription factor p53 was strongly activated following DENV infection (Fig. 2D). Inhibition of p53, using the specific inhibitor pifithrin-α was able to partially suppress DENV-induced apoptosis (Fig. 6F), as did the pan-caspase inhibitor Z-VAD-fmk in Mo-DC (Fig. 6F). Altogether, these results argue that NOX-dependent induction of ROS stimulated p53-regulated mitochondrial and caspase-dependent apoptosis.

While infected cells displayed apoptotic markers as described above, uninfected bystander Mo-DC cells did not undergo apoptosis, but rather increased expression of the differentiation and activation markers CD83 and CD86 (Fig. 6G and S6A–C Figure), CD40, CD80, CD86 and PD-L1 (S6D Figure). When cells were pre-treated with DPI prior to DENV infection, the number of CD83-positive bystander cells decreased by 2.2 fold, compared to non-treated cells (Fig. 6H). To determine if ROS production altered the antiviral response in uninfected bystander cells *via* cytokine release, conditioned media from DENV-infected DC pre-treated or not with DPI was transferred to uninfected Mo-DC (Fig. 6I). Pre-treatment with conditioned media from DPI-treated DC altered the susceptibility of naïve cells to DENV infection, as shown by the ∼2-fold increase in DENV E protein expression. Altogether, ROS contributes to mitochondria-dependent apoptosis, and also contributes to the maturation of uninfected bystander DC.

### Nrf2 transcription factor controls DENV infection and its associated immune and apoptotic response

**Figure 7:**
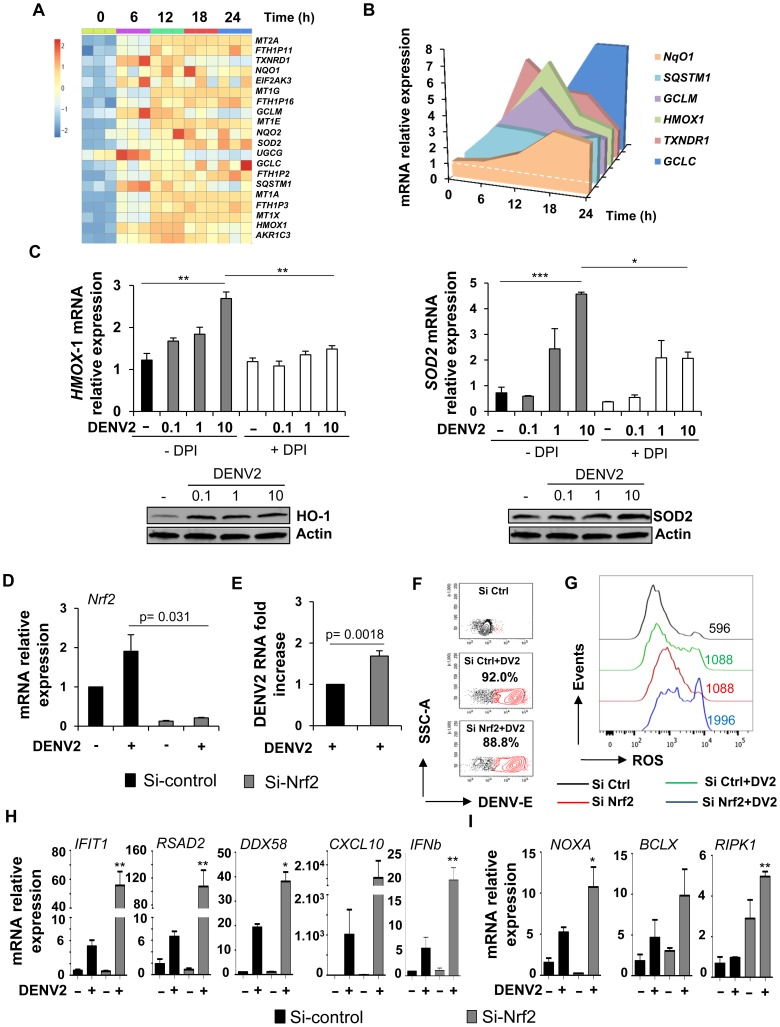
Nrf2 transcription factor limits DENV infection and modulates the innate immune and apoptotic responses. (A) Gene expression heatmap of antioxidant expressed genes modulated by dengue infection in Mo-DC at various times of infection (6–24 h) when compared to baseline. The scale shows the level of gene expression where red and blue correspond to up- and down-regulation respectively. Each box of the heatmap represents one donor. (B) The expression level of Nrf2-regulated genes was determined by qPCR at various times after DENV challenge. The data are representative of three donors. (C) Mo-DC were pre-treated with DPI (3 µM) and subsequently challenged with increasing amounts of DENV (MOI 0.1–10). The gene and protein expression levels of HMOX-1 and SOD2 were evaluated by qPCR and immunoblot, respectively. Data are the means ± SEM of one experiment performed on three individual donors. Immunoblots are representative of the results from one donor. (**D–E**) Mo-DC were transfected with control or Nrf2 siRNA and 48 h later were infected with DENV (MOI 1). Nrf2 mRNA expression level (D) and DENV viral RNA (E) were assessed by qPCR. (**F–G**) Mo-DC were transfected with Control or Nrf2 siRNA and 48 h later were infected with DENV (MOI 20). Percentage of DENV-infected cells and ROS accumulation was determined in the same samples at 18 h after infection. (H–I) mRNA expression levels of antiviral (H) and apoptotic genes (I) were measured by qPCR in Nrf2-depleted cells that were infected with DENV (MOI 1). Data are the mean ± SEM of two independent experiments performed in duplicate on two donors. P values were determined based on the comparison with DENV2-infected si-control-transfected cells.

**Figure 8:**
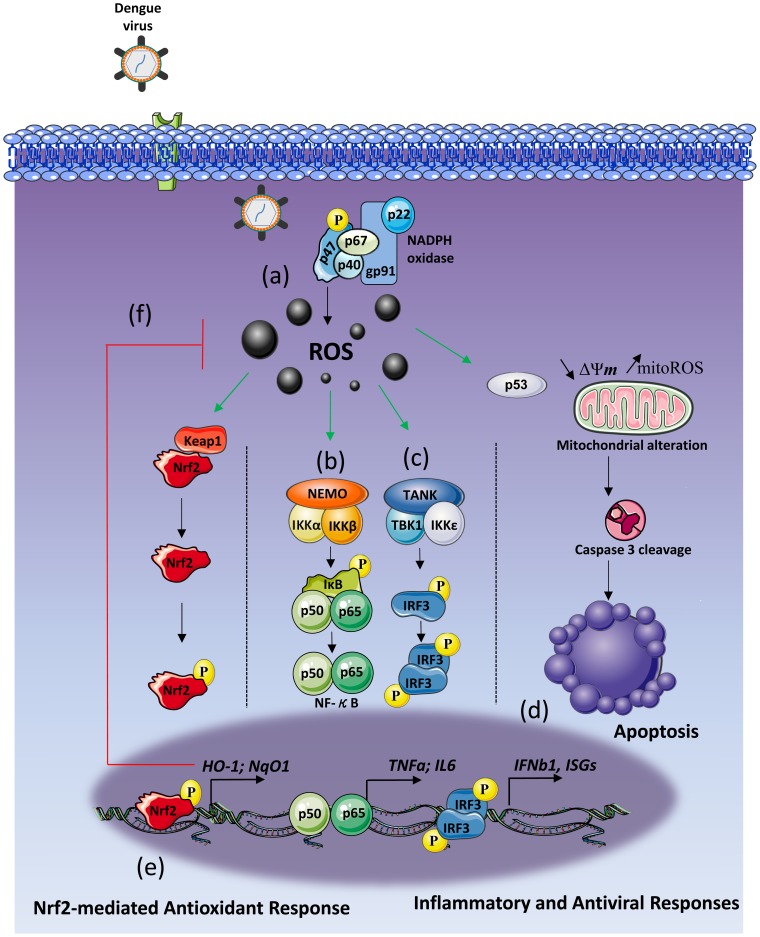
Schematic of DENV induces NOX-dependent ROS production required for antiviral and apoptotic responses. DENV2 infection generates intracellular NOX-derived ROS accumulation in Mo-DC (A). DENV2-induced ROS formation is essential for the activation of the NF-κB inflammatory response (B) and for the IRF3-mediated antiviral response (C). ROS accumulation leads to p53 stimulation which generates a mitochondrial and caspase-dependent apoptosis (D). Finally, oxidative stress generation also stimulates the cytoprotective transcription factor Nrf2 (E) which tightly regulates ROS levels (f) as well as innate immune and apoptotic responses to DENV infection.

Defense against sustained antioxidant production and the inhibition of ROS are important protective mechanisms that are regulated by the activation of Nrf2-transcription factor and downstream Nrf2-target genes. Based on the array data (Fig. 3A), Nrf2 target genes such as *HMOX-1*, *SOD2*, *NQO1*, as well as the metallothionein and ferritin families, were all rapidly stimulated by *de novo* DENV2 infection (Fig. 7A) and transient induction of these genes was confirmed by qPCR (Fig. 7B). Levels of heme-oxygenase-1 (HMOX-1) and superoxide dismutase-2 (SOD-2) mRNA were sensitive to the ROS scavenger DPI which abrogated the increase in *HMOX-1* and *SOD-2* (Fig. 7C). When Nrf2 expression was silenced using Nrf2-specific siRNA (both at the mRNA (Fig. 7D) and at the protein level (S7A Figure), decreases in the mRNA levels of Nrf2-dependent antioxidant genes were also observed (S7B Figure). Functionally, the redox homeostasis was critically affected in Nrf2-deleted Mo-DC, as shown by the ∼3 fold increase in ROS accumulation (S7C Figure). Although silencing of Nrf2 only slightly increased DENV2 RNA accumulation (Fig. 7E) and DENV infectivity (Fig. 7F) after 24 h of infection, the impairment of Nrf2 expression drastically potentiated oxidative stress response in DENV-infected cells (Fig. 7G). Indeed, a ∼2 fold increase in ROS generation was observed between DENV-infected control- and siRNA-expressing, Nrf2-transfected cells (Fig. 7G) for the same number of infected cells (Fig. 7F). Finally, the mRNA levels of genes associated with the antiviral and inflammatory response such as *IFIT1*, *RSAD2*, *DDX58*, *CXCL10* and *IFNb* (Fig. 7H), as well as genes involved in the apoptotic response such as *NOXA*, *BCLX*, and *RIPK1* (Fig. 7I) were all significantly increased. Altogether, these data demonstrate that the Nrf2-regulated antioxidant pathway is stimulated as part of the stress response after DENV infection; the Nrf2-dependent genes regulate the levels of ROS production and thus modulate the immune and apoptotic responses against DENV infection (Fig. 8).

## Discussion

Evaluation of the early host immune response to DENV infection is essential for a complete understanding of the complex immunopathogenesis associated with the development of mild or severe dengue fever in patients. Previous studies have demonstrated that DENV can trigger an innate immune response that includes the release of antiviral and inflammatory cytokines [50], [53], [54], while other studies demonstrate the ability of DENV to antagonize the induction of innate responses via cleavage of the endoplasmic reticulum adaptor STING [51], [55]. To uncover novel regulatory pathways involved in DENV infection of Mo-DC, we have for the first time used a transcriptome-wide expression analysis, coupled with biochemical dissection, to investigate the early host response to DENV infection in primary human dendritic cells - an important pool of cells infected early *in vivo* after the bite of the mosquito *Aedes aegypti*. Here, we demonstrate that: 1) DENV preferentially infected myeloid cells as they differentiated *in vitro* to mature Mo-DC; 2) DENV2 infection triggered antiviral, inflammatory, and oxidative stress pathways with distinct kinetics; 3) DENV2 infection generated a NOX-dependent intracellular accumulation of ROS; 4) ROS production mediated activation of the IRF3/STAT1- and NF-κB-mediated innate immune responses; 5) ROS production mediated p53 mitochondrial-dependent apoptosis and contributed to bystander Mo-DC maturation/activation; and 6) Nrf2-regulated target genes limited the oxidative stress response, and ultimately modulated ROS-induced immune and apoptotic responses. These results highlight a requirement for the oxidative stress response in the generation of the host innate immune response to DENV infection.

Activation of the NADPH-oxidase (NOX) complex and generation of reactive oxygen species (ROS) has been described for several viral infections, including hepatitis C virus (HCV), Rhinovirus, and HIV [56]–[58]. We demonstrate that DENV infection also activates NOX-dependent ROS production in Mo-DC. In some infection models, viral proteins such as Nef and Tat for HIV and NS3 for HCV were shown to specifically stimulate the NOX complex [59]–[61]. NOX activity was also regulated by spleen tyrosine kinase (Syk)-mediated phosphorylation of the NOX p47phox subunit [62]; Syk kinase is downstream of the surface receptor CLEC5A, which was shown to promote inflammasome activation and inflammatory cytokine release in DENV infection [63], [64]. Importantly, TLR3, a receptor critically involved in RNA sensing, was recently shown to stimulate NOX-dependent ROS production that was required for NF-κB, IRF3 and STAT1 activation in murine macrophages in response to the synthetic dsRNA Poly (I:C) [65]. Furthermore, exogenous addition of oxidative stress potentiated the TLR3 response to dsRNA in airway epithelial cells [66]. Finally, the specific TLR7 agonist imiquimod also elevated basal superoxide production through enhanced NOX2 activity in macrophages [67]. Further studies are now required to determine the exact mechanism(s) involved in DENV-induced NOX-dependent ROS production in human Mo-DC.

ROS were long considered as toxic, microbe-induced by-products involved in the killing of pathogens [18]; however, their function as second messengers that regulate immune signaling suggests a much broader role in host defense against viruses [19]–[23]. ROS production was in fact required to trigger the antiviral and inflammatory responses to DENV infection in DC, and was confirmed by both chemical and genetic inhibition of the NOX complex. Blockade of NOX activation or ROS production inhibited antiviral and inflammatory responses, including the IRF3/STAT1 antiviral axis and the NF-κB inflammatory pathway (Fig. 4). The IRF3 pathway has previously been demonstrated to be regulated by oxidative stress variations. Indeed, the expression level of the non-canonical IKK-like kinase, IKKε, is itsef NOX-regulated and participated in the immune response induced by the respiratory syncytial virus (RSV) [68]. NOX-derived ROS were also shown to activate the RIG-I/MAVS/IRF3 antiviral axis in epithelial cells, and were required to maintain the constitutive level of MAVS expression [22]. In contrast, statistical changes in MAVS or IKKε expression following NOX inhibition in primary DENV-infected DC were not observed in this study (S8A–C Figure), suggesting that DENV-induced ROS may regulate host response *via* post-translational modification of proteins involved in antiviral signaling, as was described previously for S-glutathionylation of TRAF3 and TRAF6 [19]. Other non-infectious biological processes such as impairment of autophagy also support the idea that oxidative stress modulates the sensitivity to antiviral signaling. Indeed, blocking of autophagy allows for oxidative stress accumulation through defective mitochondria and leads to the amplification of RLR signaling [69]. Altogether, these studies cumulatively highlight the complexity of ROS involvement in the stimulation of antiviral responses and argues that the innate immune response integrates both viral RNA sensing and detection of homeostatic perturbations to coordinate an appropriate host response.

The Nrf2-mediated antioxidant response was one of the top differentially regulated pathways early after DENV infection, resulting in the expression of many cytoprotective enzymes such as HMOX-1, SOD2, NQO1, GCLC and GCLM, that function together to maintain an appropriate redox status, and thus protect cells from ROS-induced damage [24]–[26]. The importance of Nrf2 activity during viral pathogenesis was demonstrated recently in a study showing that Marburg virus (MARV) hijacked the Nrf2 pathway leading to a persistent activation of Nrf2-dependent antioxidant and cytoprotective genes, temporarily blocking cell death of MARV-infected cells, and thus facilitating viral proliferation [70], [71]. Another study involving Nrf2 knockout mice demonstrated that mice challenged with Respiratory Syncytial Virus (RSV) or influenza had both higher viral replication and increased inflammatory responses and injury in their lungs [34], [72], [73]. Consistent with these observations, genetic silencing of Nrf2 in primary Mo-DC deregulated intracellular redox homeostasis and led to increased inflammatory and apoptotic responses. The importance of Nrf2 in DENV pathogenesis was more recently illustrated in a study of DENV-infected HepG2 xenografted SCID mice treated with the tripeptide glutathione (GSH), an anti-oxidant whose intracellular levels are also regulated by Nrf2. GSH prevented DENV-induced oxidative stress and liver injury by inhibiting pro-inflammatory cytokine production [40]. The same observation was made *in vitro* where treatment of DENV-infected HepG2 cells with GSH prevented the increase in ROS accumulation. Administration of antioxidant molecules such as GSH or other Nrf2 activators may be a novel strategy to treat and limit symptoms associated with DENV disease.

DC are potent antigen presenting cells that, after sensing of pathogens, migrate from peripheral tissues to the lymph nodes and drive CD4+ and CD8+ T cell responses [74]. Here, we demonstrate that DENV-infected Mo-DC undergo mitochondria-dependent apoptosis, driven by an increase in ROS and facilitated by p53 transcription factor. Uninfected bystander DC, on the other hand, are not killed but rather mature to DC expressing maturation and activation markers, as previously reported [50]. ROS exposure and the immune response generated in infected cells, rendered the bystander uninfected DC less susceptible to DENV replication, most probably as a consequence of released soluble factors from infected cells. Meanwhile inhibition of ROS with DPI decreased expression of maturation markers and increased susceptibility to DENV infection. Thus, ROS production may not only impact infected cells but also affect DC maturation indirectly, by altering the cytokine milieu of uninfected bystander DC; in turn DC maturation in context of DENV infection may alter priming of the T cell response.

There are no diagnostic markers presently available that will determine whether a DENV-infected patient will develop a mild illness or progress to a more severe dengue fever, associated with DENV-induced hemorrhagic fever or shock syndrome. However, markers of oxidative stress have been reported in patients with severe DENV infection, suggesting a relationship between oxidative stress and viral pathogenesis in patients [41], [43], [44]. Soundravally *et al* demonstrated an association between the induction of proinflammatory cytokines and the levels of lipid peroxidation in patients [43]. Earlier studies also demonstrated that DENV-infected Mo-DC overproduce matrix metalloproteinase-9 (MMP-9), a result also suggested by our array analysis (Fig. 2B). The induction of MMP-9 by DENV-infected Mo-DC enhanced endothelial permeability *in vitro* and was proposed as a marker for disease severity [75]. Interestingly, increased oxidative species through NADPH-oxidase activation or upon TLR3 ligation were also shown to regulate MMP-9 expression [30], [76], [77]. Furthermore, mice lacking the p47 NADPH-oxidase subunit displayed a reduction in hemorrhage development and disease severity after DENV infection [78]. Altogether, these findings highlight a key role for NADPH-oxidase in the oxidative stress-related pathology of DENV, and suggest that both NADPH-oxidase activity, ROS levels or associated ROS-induced molecules may be useful biomarkers to predict disease severity.

In conclusion, DENV infection of DC induces intracellular ROS levels that regulate the magnitude of the activation of innate antiviral immune responses and stimulate apoptosis. Parallel activation of antioxidant pathways regulated by Nrf2 also contributes to the regulatory control of antiviral and apoptotic responses by maintaining redox homeostasis. ROS were identified as an essential component of the host response to DENV infection; a further understanding of the molecular details underlying the biological targets of ROS during DENV infection may facilitate identification of novel treatment strategies for dengue-associated diseases.

## Materials and Methods

### Ethics statement

Human peripheral blood mononuclear cells (PBMC) were isolated from buffy coats of healthy, seronegative volunteers in a study approved by the IRB and by the VGTI-FL Institutional Biosafety Committee (2011-6-JH1). Written informed consent approved by the VGTI-FL Inc. ethics review board (FWA#161) was provided to study participants. Research conformed to ethical guidelines established by the ethics committee of the OHSU VGTI and Martin Health System.

### Monocyte isolation and differentiation into Mo-DC

Briefly, PBMC were isolated from freshly collected blood using the Ficoll-Paque PLUS medium (GE Healthcare Bio) as per manufacturer's instructions. CD14^+^ monocytes were isolated by positive selection using CD14 microbeads and a magnetic cells separator as per kit instructions (Miltenyi Biotech). Purified CD14^+^ monocytes were cultured for 7 days either in six-well plates (1.5×10^6^ cells) or 100 mm dishes (15×10^6^ cells) in 2 mL (6-well plate) or 10 mL (100 mm dish), respectively of complete Mo-DC differentiation medium (Miltenyi Biotech.). On day 3, the medium was replenished with fresh medium. Purity of CD14^−^ CD1a^+^ DC-SIGN ^high^ moDC was typically >80%.

### Virus production, quantification and Mo-DC infection

DENV serotype 2 (DENV2) strain New Guinea C (DENV NGC) or DENV2 strain 16681 were produced on C6/36 cells and quantified on Vero cells as previously reported [79]. In control experiments, virus was inactivated using formalin 0.05% in PBS at 22°C or UV-inactivated for 1 h on ice. For infection, except where indicated, immature Mo-DC were infected at a multiplicity of infection of 20 in a small volume of medium without FBS for 3 hours at 37°C. Following adsorption, cells were washed twice in serum-free medium and incubated with complete medium containing cytokines prior to analysis. Mock-infected Mo-DC were treated according to the same procedure. All procedures with live DENV2 were performed in a Biosafety level 2+ facility at the Vaccine and Gene Therapy Institute of Florida.

### Microarray analysis

The DENV2 kinetics microarray experiment was performed as a single experiment on Mo-DC derived from 3 independent healthy donors. Mo-DC were infected at an MOI of 20 as described above and cells were collected at various times and lysed using RLT lysis buffer (Qiagen) for RNA extraction. Briefly, RNA were extracted using RNeasy Micro Kits (Qiagen). The quantity and the quality of the RNA were validated using a NanoDrop 2000c (Thermo Fisher). Samples were then amplified using Illumina TotalPrep RNA amplification kits (Ambion). The microarray analysis was conducted using 750 ng of biotinylated complementary RNA hybridized to HumanHT-12_V4 BeadChips (Illumina) at 58°C for 20 hours. The data were collected with Illumina GenomeStudio software. First, arrays displaying unusually low median intensity, low variability, or low correlation relative to the bulk of the arrays were discarded from the rest of the analysis. Quantile normalization, followed by a log2 transformation using the Bioconductor package LIMMA was applied to process microarrays. Missing values were imputed with the R package (http://cran.r-project.org/web/packages/impute/index.html). In order to identify differentially expressed genes between uninfected and infected samples, the LIMMA package from Bioconductor was used. For data mining and functional analyses, genes that satisfied a *p* value (<0.05) with ≥1.3 fold change (up or down) were selected. Probes that do not map to annotated RefSeq genes and control probes were removed. The expected proportions of false positives (FDR) were estimated from the unadjusted *p* value using the Benjamini and Hochberg method.

All network analysis was done with Ingenuity Pathway Analysis (IPA: Ingenuity systems). The differentially expressed genes selected based on above criteria were mapped to the ingenuity pathway knowledge base with different colors. The significance of the association between the dataset and the canonical pathway was measured in two ways: (1) A ratio of the number of genes from the dataset that map to the pathway divided by the total number of genes that map to the canonical pathway was displayed; (2) by over-representation analysis Fisher's exact test was used to calculate a *p*-value determining the probability that the association between the genes in the dataset and the canonical pathway is explained by chance alone. The pathways were ranked with −log *p*-values. The pathway enrichment and network analyses were done using Ingenuity Pathway Analysis (IPA: Ingenuity systems). The differentially expressed genes were further selected based on p-value (0.001) and subsequently were mapped to the Ingenuity Pathway knowledgebase. The significance of the association between the dataset and the canonical pathway was measured in two ways: (1) A ratio of the number of genes from the dataset that map to the pathway divided by the total number of genes that map to the canonical pathway was displayed; (2) by overrepresentation analysis: Fisher's exact test was used to calculate a p-value determining the probability that the association between the genes in the dataset and the canonical pathway is explained by chance alone. The top ranking pathways were selected by ranking −log p-values. The selected pathways were then represented as networks by grouping genes involved in a pathway as a cloud by retaining the relationships represented as edges. Manual curation was further employed to annotate selected pathways by adding genes and their relationships to other genes in networks that are not depicted by Ingenuity. Subsequently, genes were color-coded based on the fold-changes (green – downregulated; red – upregulated). Heatmaps of these genes were generated to display both fold-changes and membership of genes in one or more pathways; these heatmaps were created using the R statistical computing environment. The data have been deposited in the NCBI Gene Expression Omnibus (GEO Series accession number GSE58278).

### Quantitative real-time PCR

Total RNA was isolated from cells using RNeasy Kit (Qiagen) as per manufacturer's instructions. RNA was reverse transcribed using the SuperScript VILO cDNA synthesis kit according to manufacturer's instructions (Invitrogen). PCR primers were designed using Roche's Universal Probe Library Assay Design Center (www.universalprobelibrary.com). Quantitative RT-PCR was performed on a LightCycler 480 system using LightCycler 480 Probes Master (Roche). The N-fold differential expression of mRNA gene expression was expressed as 2^−ΔΔCt^.

### Fluidigm BioMark assay

The DENV2 kinetics BioMark experiment was performed with Mo-DC derived from 3 independent healthy donors. Total RNA and cDNA were prepared as described above. Intron-spanning PCR primers were designed using Roche's Universal Probe Library Assay Design Center (www.universalprobelibrary.com) and obtained from the Integrated DNA Technology company (USA) (S1 Table). cDNA along with the entire pool of primers were pre-amplified for 14 cycles using TaqMan PreAmp Master Mix as per manufacturer's protocol (Applied Biosystems). cDNA was treated with Exonuclease I (New England Biolabs). cDNA samples were prepared with 2X FastStart TaqMan Probe Master (Roche), GE sample loading buffer (Fluidigm) and Taq Polymerase (Invitrogen). Assays were prepared with 2X assay loading reagent (Fluidigm), primers (IDT) and probes (Roche). Samples and assays were loaded in their appropriate inlets on a 48.48 BioMark chip. The chip was run on the BioMark HD System (Fluidigm), which enabled quantitative measurement of up to 48 different mRNAs in 48 samples under identical reaction conditions. Runs were 40 cycles. Raw Ct values were calculated by the real time PCR analysis software (Fluidigm) and software-designated failed reactions were discarded from analysis. All data are presented as a relative quantification with efficiency correction based on the relative expression of target gene versus the geomean of (GAPDH+Actin+β2 microglobulin) as the invariant control. The N-fold differential expression of mRNA gene samples was expressed as 2^−ΔΔCt^. The heatmaps were produced with the following package; pheatmap: Pretty Heatmaps. R package version 0.7.7 http://CRAN.R-project.org/package=pheatmap. Gene level expression is shown as −ΔΔCt or gene-wise standardized expression (Z score). The sequences of primers used as well as their complementary probes are listed in the S1 Table.

### Flow cytometry analyses

#### Surface staining

Before staining, FcγR were blocked using the Human TruStain FcX Solution (BioLegend) for 10 min at room temperature in PBS 2% FBS. Cells were then stained for 15 min at 4°C in PBS 2% FBS with one or more of the following Ab: anti- CD14-AF700 (BioLegend), anti-DC-SIGN-AF647 (BioLegend), anti-CD1a-AF488 (BioLegend), anti-CD83-PE (BioLegend), anti-CD86-Pacific Blue (BioLegend), anti-CD40 PE (BioLegend), anti-CD80 Pacific Blue (BD Biosciences), anti-PD-L1 APC (BioLegend), anti HLA-DR A700 (BioLegend).

#### DENV E staining

The percentage of cells infected with DENV was determined by intracellular staining using a mouse IgG2a mAb, specific for DENV E protein (clone 4G2) as previously described [79].

#### ROS production

Total ROS production using the CM-H2DCFDA probe (Life Technologies) (1 µM) or was evaluated by flow cytometry. Following DENV infection, cells were washed in PBS before incubation with the probes for 30 min at 37°C. After incubation, cells were washed twice in PBS before FACS analysis.

#### PhosFlow

PhosFlow stainings were all performed in a 96-well plate format. Cells were resuspended in 100 µL of PBS and fixed with the same volume of pre-warmed Fix Buffer I (BD Biosciences) for 10 min at 37°C. Cells were pelleted down by centrifugation and resuspended in 200 µL ice-cold PERM BUFFER III (BD Biosciences) for 20 min at 4°C. Cells were then washed three times with 200 µL of PBS containing 5% FBS and then incubated for 30 min on ice in PBS 2% FBS. Cells were pelleted down by centrifugation and stained in 50 µL PBS 2%FBS with the P-STAT1 Y701 Pacific Blue antibody (BD Biosciences) for 30 min at room temperature. Cells were finally washed twice in staining buffer and analyzed by flow cytometry. In all the proposed flow cytometry experiments, cells were analyzed on a LSRII flow cytometer (Becton Dickinson, New Jersey, USA). Calculations, compensations as well as population analyses were done using FACS Diva software and overlay representations were done using FlowJo.

### Protein extraction and immunoblot analysis

Protein lysates (20 to 40 µg) from Mo-DC were subjected to western blot analysis. Membranes were probed with primary antibodies: anti-pIRF3 at Ser 396 (EMD Millipore), anti-IRF3 (IBL, Japan), anti-IRF7 (EMD Millipore), anti-RIG-I (EMD Millipore), anti-IFIT1 (Thermo Fisher Scientic), anti pSTAT1 at Tyr701 (Cell Signaling), anti-STAT1 (Cell Signaling), anti–pIκBα at Ser32 (Cell Signaling), anti-IκBα (Cell Signaling), anti-p47phox at Ser359 (AssayBioTech), anti-p47phox (Sigma Aldrich), anti-STING (Cell Signaling), anti-gp91phox (Santacruz Biotechnology), anti-Nrf2 (Cell Signaling), anti-β-actin (Odyssey, USA). Antibody signals were detected by immunofluorescence using the IRDye 800CW and IRDye 680RD secondary antibodies (Odyssey, USA) and the LI-COR imager (Odyssey, USA). Protein expression levels were determined and normalized to β-actin using the ImageJ software (National Institutes of Health, Bethesda, USA).

### Cytometric bead array

Cytokine production was evaluated in the supernatants of DENV2-infected Mo-DC using a BD CBA flex set (IFN-α, TNF-α, IL-6, IL-1β, IL-10, IL-12p70) as per manufacturer's recommendations. The BD FACS Array Bioanalyzer was used to process the samples and perform the analysis.

### Small interfering RNA assays

Two different methods were used for siRNA transfection of Mo-DC. A total of 3×10^6^ Mo-DC were transfected in a cuvette in the presence of 100 pmol of control (sc-37007), Nrf2 (sc-37030) or gp91-phox (sc-35503) human siRNA (Santa Cruz Biotechonlogy, USA) using the Amaxa 4D-Nucleofector Technology for 48 h. The Amaxa P3 Primary Cell 4D Nucleofector X Kit was used with the electroporation program EA-100. Another method based on a transfection reagent was alternatively used to transfect lower amount of cells. A total of 4×10^5^ Mo-DC was transfected in 24-well plates in the presence of 40 pmol of control (sc-37007), Nrf2 (sc-37030), gp91-phox (sc-35503) human siRNA (Santa Cruz Biotechonlogy, USA) using 6 µL of HiPerfect Transfection Reagent (Qiagen) for 48 h.

### Statistical analysis

Values were expressed as the mean ± SEM and statistical analysis, except where indicated, was performed with Microsoft Excel or Graph Pad Prism, using an unpaired, two-tailed Student's *t* test to determine significance. *P* values of less than 0.05 were considered statistically significant, ***, *p*<0.001; **, *p*<0.01, and *, *p*<0.05.

## Supporting Information

S1 Figure **High throughput analysis of DENV2-associated host response in Mo-DC.** Mo-DC from 3 individual donors were infected with DENV2 (MOI 20) and sampled at 0–24 h post-infection. (A) Heatmap of the three donor gene profiles after DENV2 infection, evaluated by high throughput qPCR. Gene expression levels are represented by −ΔΔCt values where red corresponds to an up- regulation and blue to a down-regulation of gene expression. (B) Kinetics of selected genes from one donor grouped by function. (C) Correlation between the Log FC values of gene expression from the microarray and from the high throughput qPCR experiment was calculated (n = 49; Spearman test).
(TIF)

S2 Figure **Genes and pathways from cloud map analysis displayed in a heatmap.** Log (base2) fold-changes are represented by intensity of red and green (for up- and down-regulated genes, respectively). Genes are ranked by fold-change in each row, unless included in earlier rows. A neutral color indicates that a gene does not belong to a given pathway. (A) 6 h and (B) 24 h.
(TIF)

S3 Figure **Cytotoxic effect of antioxidant molecules on Mo-DC.** (A) Summary table of the working concentrations as well as the mode of action of the antioxidant molecules used in the study. (B–C) Mo-DC were treated for 24 h with the different antioxidant molecules cited in (A). Cell viability was assessed by flow cytometry using an Annexin-V and 7AAD staining cocktail.
(TIF)

S4 Figure **NOX-inhibitor increases DENV RNA accumulation.** Mo-DC were pretreated with the NOX inhibitor DPI (0.3–3–30 µM) for 1 h and subsequently infected with DENV2 (MOI 20). DENV RNA accumulation was detected by qPCR. Data are the means ± SEM of one experiment performed on three individual donors.
(TIF)

S5 Figure **DENV-induced immune responses require a replicating dengue virus.** (A–B) Formalin-inactivated (A) or UV-inactivated (B) DENV was used to challenge Mo-DC for 24 h (MOI 20). (i) viral RNA (qPCR), (ii) percentage of DENV-infected cells (FACS) and (iii) gene expression level (PCR) was detected 24 h after inactive-DENV2 challenge. The data are the means of one experiment performed in triplicate. P values were determined based on the comparison with live DENV2-infected cells. (C) Gene expression level of various antiviral genes was detected in Mo-DC pre-treated with the NADPH-oxidase inhibitor DPI (1 µM) for 1 h and challenged with DENV2 (strain NGC) (MOI 20) or DENV2 (strain 16681). Data are for one representative experiment performed in triplicate. P values were determined based on the comparison with the appropriate DENV2-infected control.
(TIF)

S6 Figure **DENV infection activates uninfected bystander cells.** (A–C) Mo-DC were infected with DENV (MOI 1 or 20) or treated with LPS (1 µg/mL) for 48 h. CD83 and CD86 protein expression levels were evaluated by FACS among the infected Annexin-V+ cells or uninfected Annxexin-V− cells. (D) Phenotypic profile of the Annexin-V− cell population (so-called bystander cell population), 48 h after DENV challenge at MOI 1.
(TIF)

S7 Figure **Efficiency of Nrf2 silencing in Mo-DC.** Mo-DC were transfected with control or Nrf2 siRNA for 48 h. (A) Nrf2 protein level was determined by immunoblot. (B) Nrf2 and antioxidant/detoxifying enzymes gene levels were determined by qPCR. (C) ROS levels were determined by FACS using the H2DCFDA fluorescent probe. Results from two independent donors are represented.
(TIF)

S8 Figure **Chemical blocking of NOX affects neither MAVS nor IKKε expression.** Mo-DC were pre-treated with the NOX inhibitor DPI (0.3–3 µM) for 1 h and subsequently infected with DENV2 (MOI 20). IKKε mRNA (A), MAVS mRNA (B) and MAVS protein (C) expression levels were detected by qPCR or immunoblotting, respectively. For panels (A) and (B), the data are the means ± SEM of one experiment performed in triplicate on three individual donors. For panel (C), the experiment has been performed on one donor.
(TIF)

S1 Table **Primer sequences and probes used for the high throughput qPCR analysis.**
(DOCX)
