# Supplementary figures and images for: Cellular Oxidative Stress Response Controls the Antiviral and Apoptotic Programs in Dengue Virus-Infected Dendritic Cells
Source: PLoS Pathog. 2014 Dec 18;10(12):e1004566. doi: 10.1371/journal.ppat.1004566 (PMC4270780; doi:10.1371/journal.ppat.1004566)

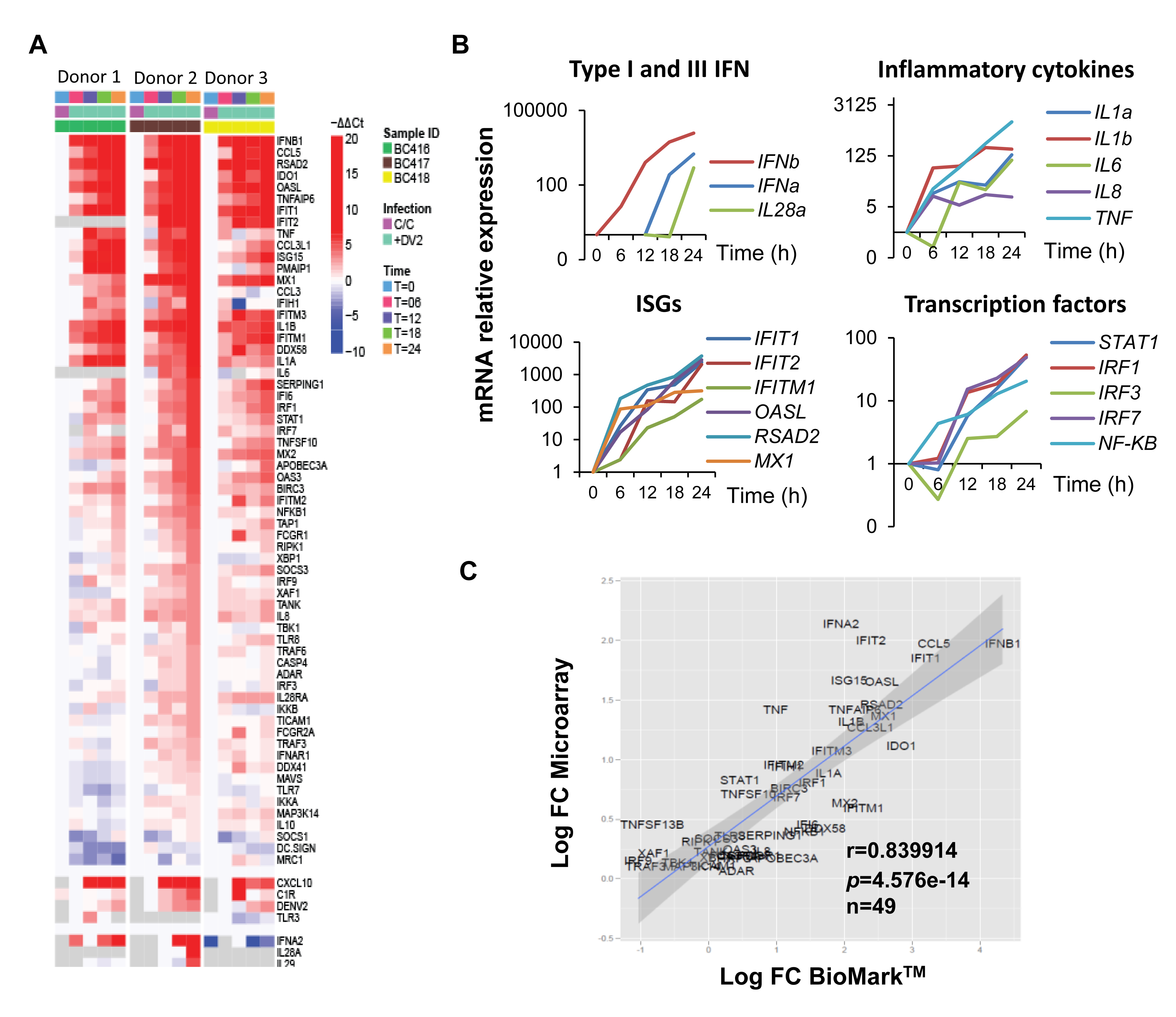

Supplement: S1 Figure — High throughput analysis of DENV2-associated host response in Mo-DC. Mo-DC from 3 individual donors were infected with DENV2 (MOI 20) and sampled at 0–24 h post-infection. (A) Heatmap of the three donor gene profiles after DENV2 infection, evaluated by high throughput qPCR. Gene expression levels are represented by −ΔΔCt values where red corresponds to an up- regulation and blue to a down-regulation of gene expression. (B) Kinetics of selected genes from one donor grouped by function. (C) Correlation between the Log FC values of gene expression from the microarray and from the high throughput qPCR experiment was calculated (n = 49; Spearman test). (TIF) [file ppat.1004566.s001.tif]

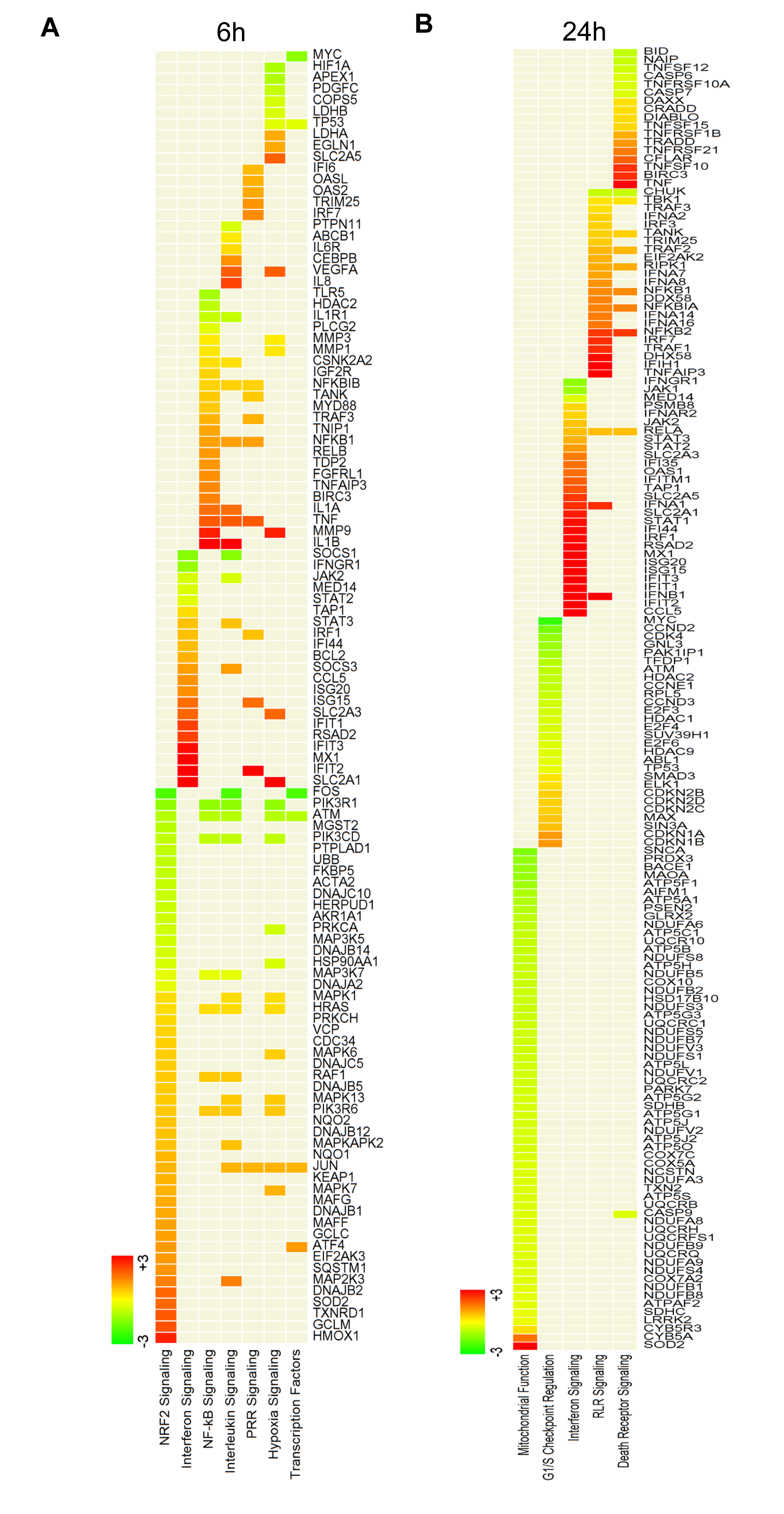

Supplement: S2 Figure — Genes and pathways from cloud map analysis displayed in a heatmap. Log (base2) fold-changes are represented by intensity of red and green (for up- and down-regulated genes, respectively). Genes are ranked by fold-change in each row, unless included in earlier rows. A neutral color indicates that a gene does not belong to a given pathway. (A) 6 h and (B) 24 h. (TIF) [file ppat.1004566.s002.tif]

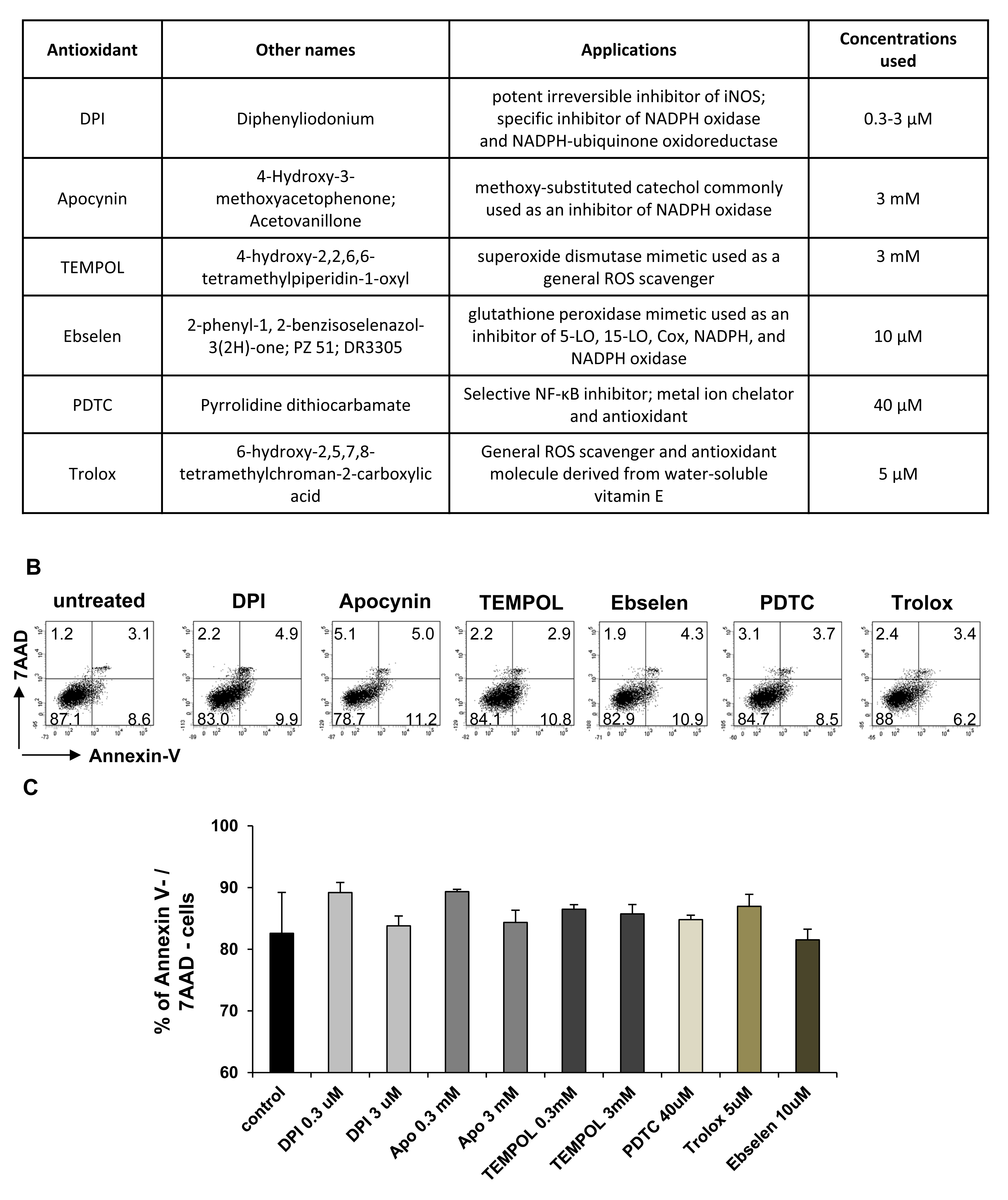

Supplement: S3 Figure — Cytotoxic effect of antioxidant molecules on Mo-DC. (A) Summary table of the working concentrations as well as the mode of action of the antioxidant molecules used in the study. (B–C) Mo-DC were treated for 24 h with the different antioxidant molecules cited in (A). Cell viability was assessed by flow cytometry using an Annexin-V and 7AAD staining cocktail. (TIF) [file ppat.1004566.s003.tif]

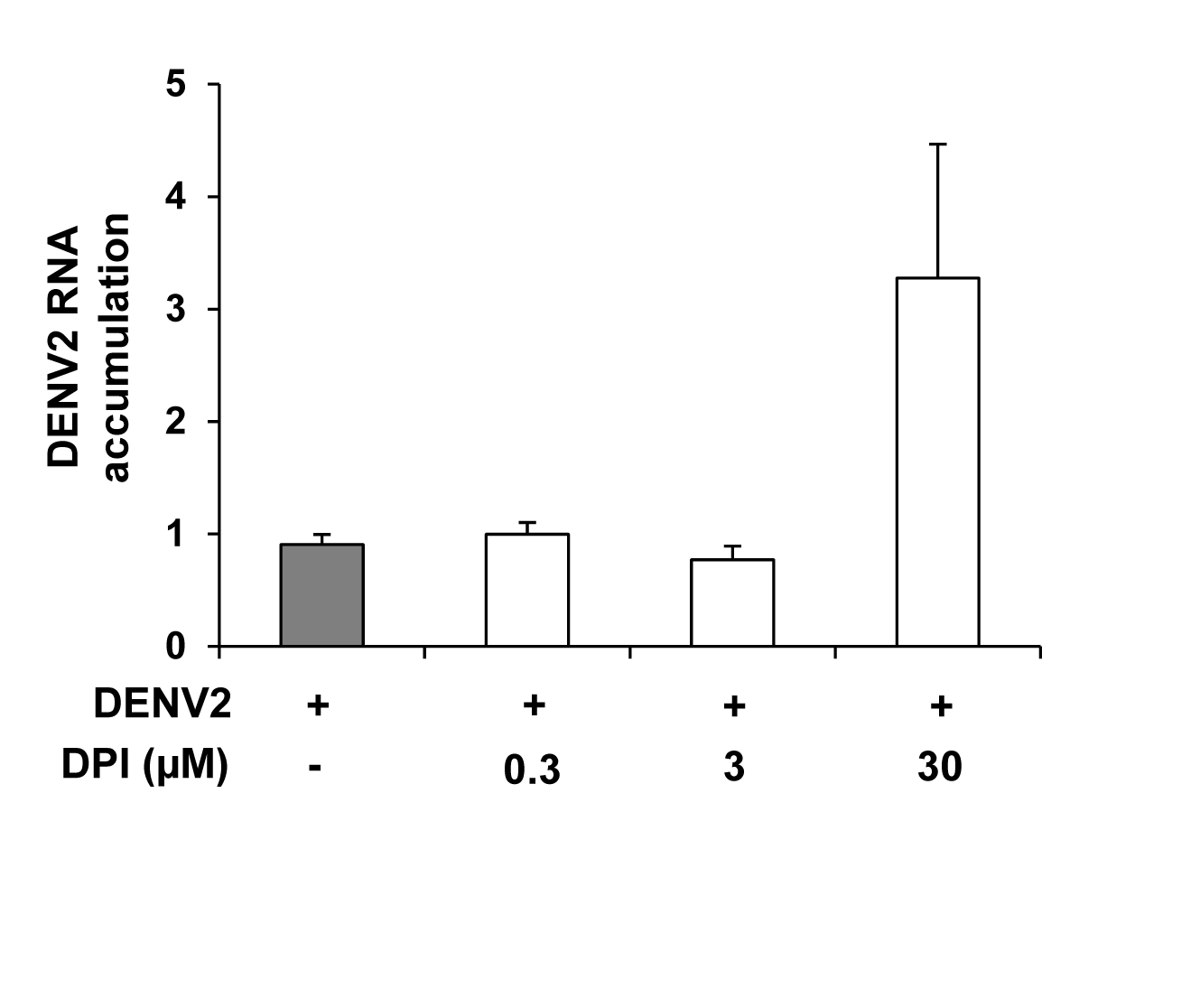

Supplement: S4 Figure — NOX-inhibitor increases DENV RNA accumulation. Mo-DC were pretreated with the NOX inhibitor DPI (0.3–3–30 µM) for 1 h and subsequently infected with DENV2 (MOI 20). DENV RNA accumulation was detected by qPCR. Data are the means ± SEM of one experiment performed on three individual donors. (TIF) [file ppat.1004566.s004.tif]

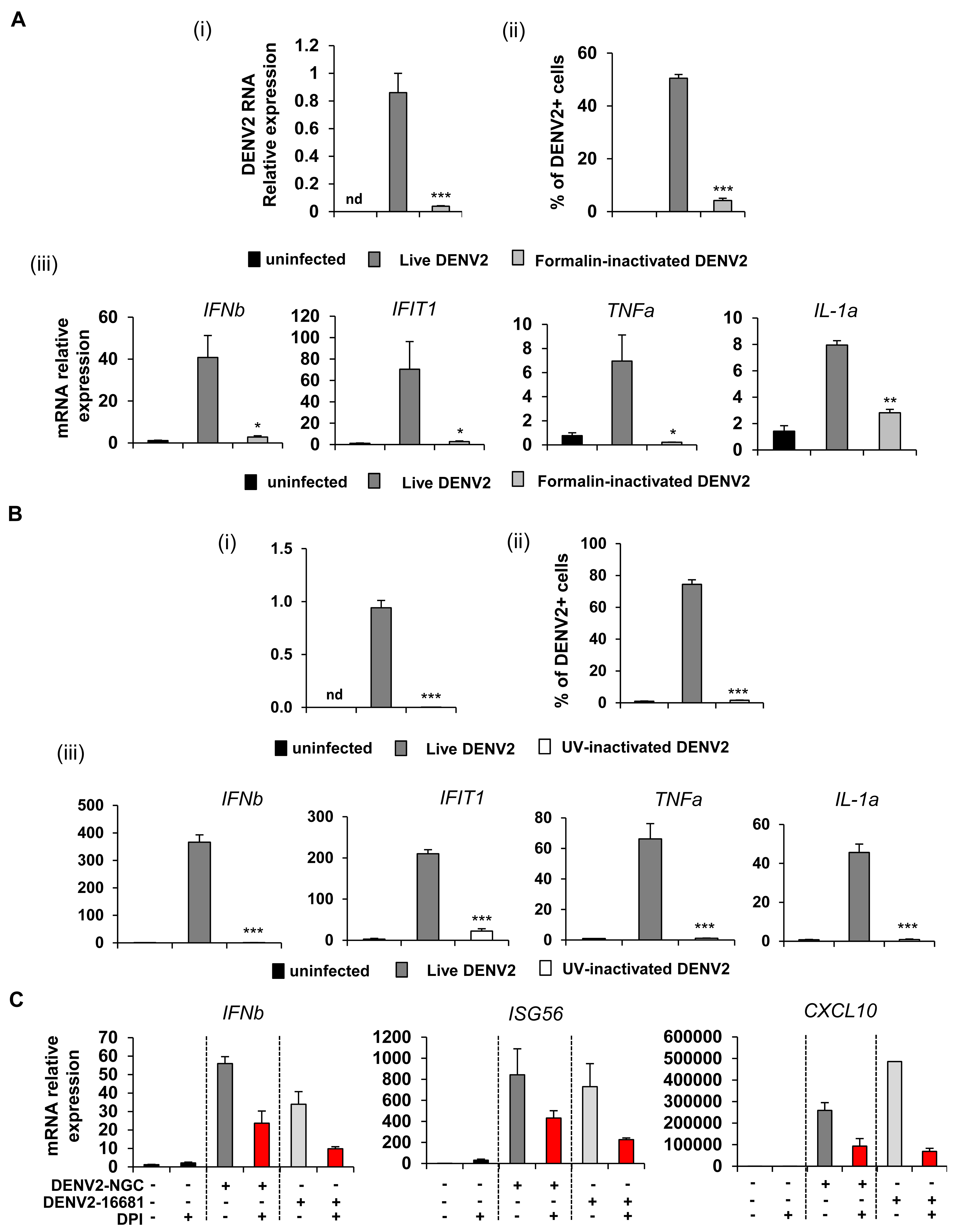

Supplement: S5 Figure — DENV-induced immune responses require a replicating dengue virus. (A–B) Formalin-inactivated (A) or UV-inactivated (B) DENV was used to challenge Mo-DC for 24 h (MOI 20). (i) viral RNA (qPCR), (ii) percentage of DENV-infected cells (FACS) and (iii) gene expression level (PCR) was detected 24 h after inactive-DENV2 challenge. The data are the means of one experiment performed in triplicate. P values were determined based on the comparison with live DENV2-infected cells. (C) Gene expression level of various antiviral genes was detected in Mo-DC pre-treated with the NADPH-oxidase inhibitor DPI (1 µM) for 1 h and challenged with DENV2 (strain NGC) (MOI 20) or DENV2 (strain 16681). Data are for one representative experiment performed in triplicate. P values were determined based on the comparison with the appropriate DENV2-infected control. (TIF) [file ppat.1004566.s005.tif]

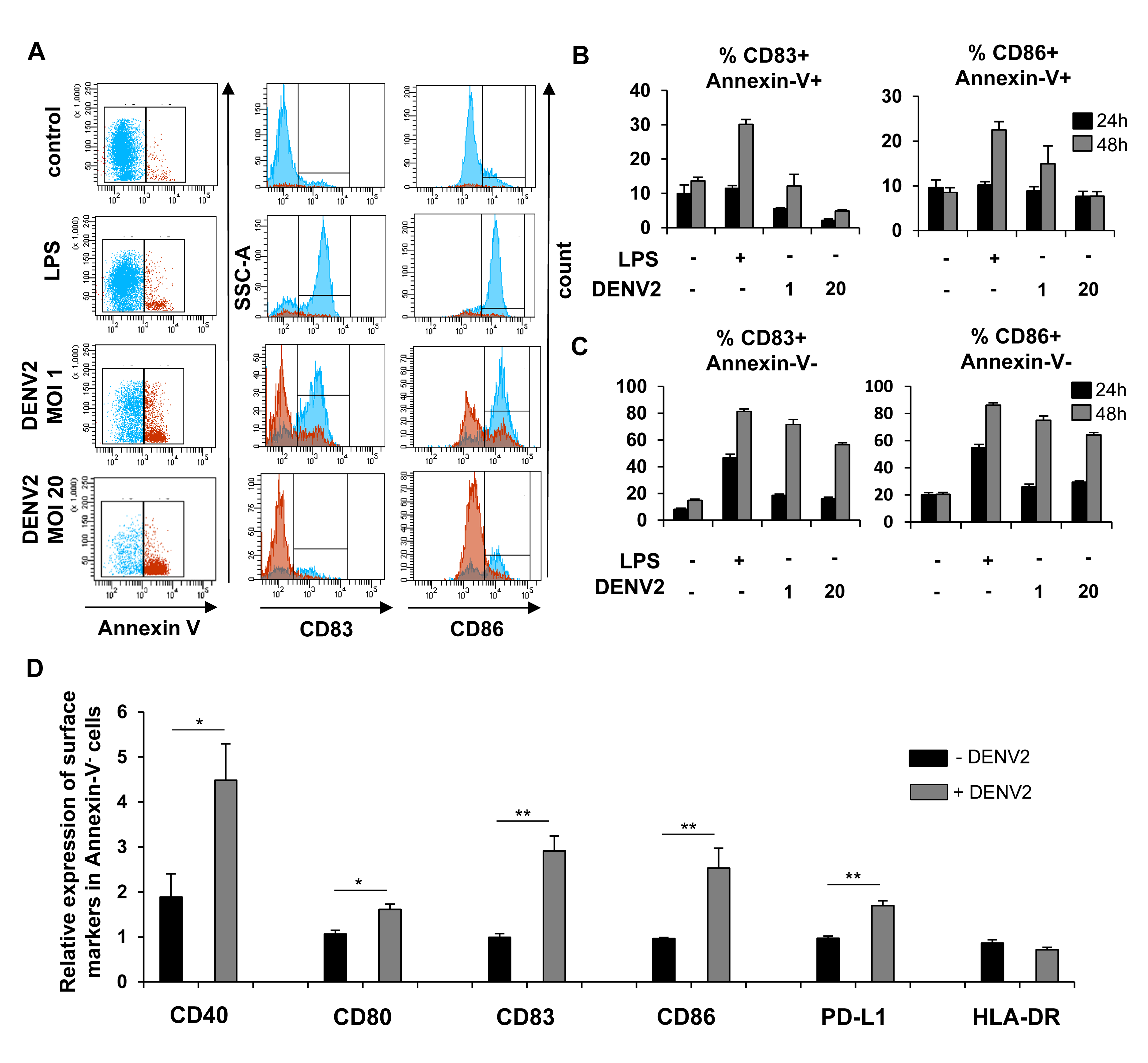

Supplement: S6 Figure — DENV infection activates uninfected bystander cells. (A–C) Mo-DC were infected with DENV (MOI 1 or 20) or treated with LPS (1 µg/mL) for 48 h. CD83 and CD86 protein expression levels were evaluated by FACS among the infected Annexin-V+ cells or uninfected Annxexin-V− cells. (D) Phenotypic profile of the Annexin-V− cell population (so-called bystander cell population), 48 h after DENV challenge at MOI 1. (TIF) [file ppat.1004566.s006.tif]

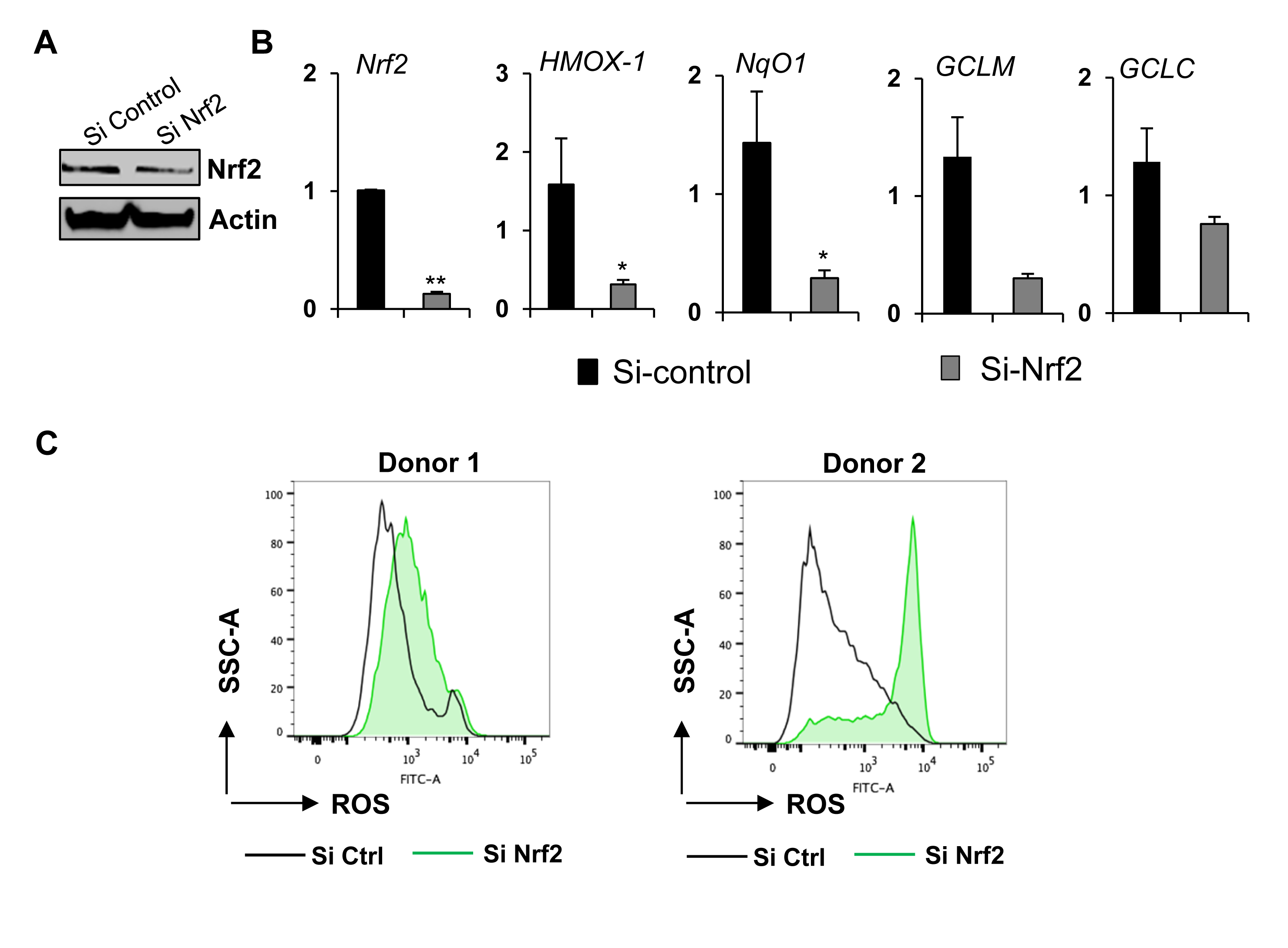

Supplement: S7 Figure — Efficiency of Nrf2 silencing in Mo-DC. Mo-DC were transfected with control or Nrf2 siRNA for 48 h. (A) Nrf2 protein level was determined by immunoblot. (B) Nrf2 and antioxidant/detoxifying enzymes gene levels were determined by qPCR. (C) ROS levels were determined by FACS using the H2DCFDA fluorescent probe. Results from two independent donors are represented. (TIF) [file ppat.1004566.s007.tif]

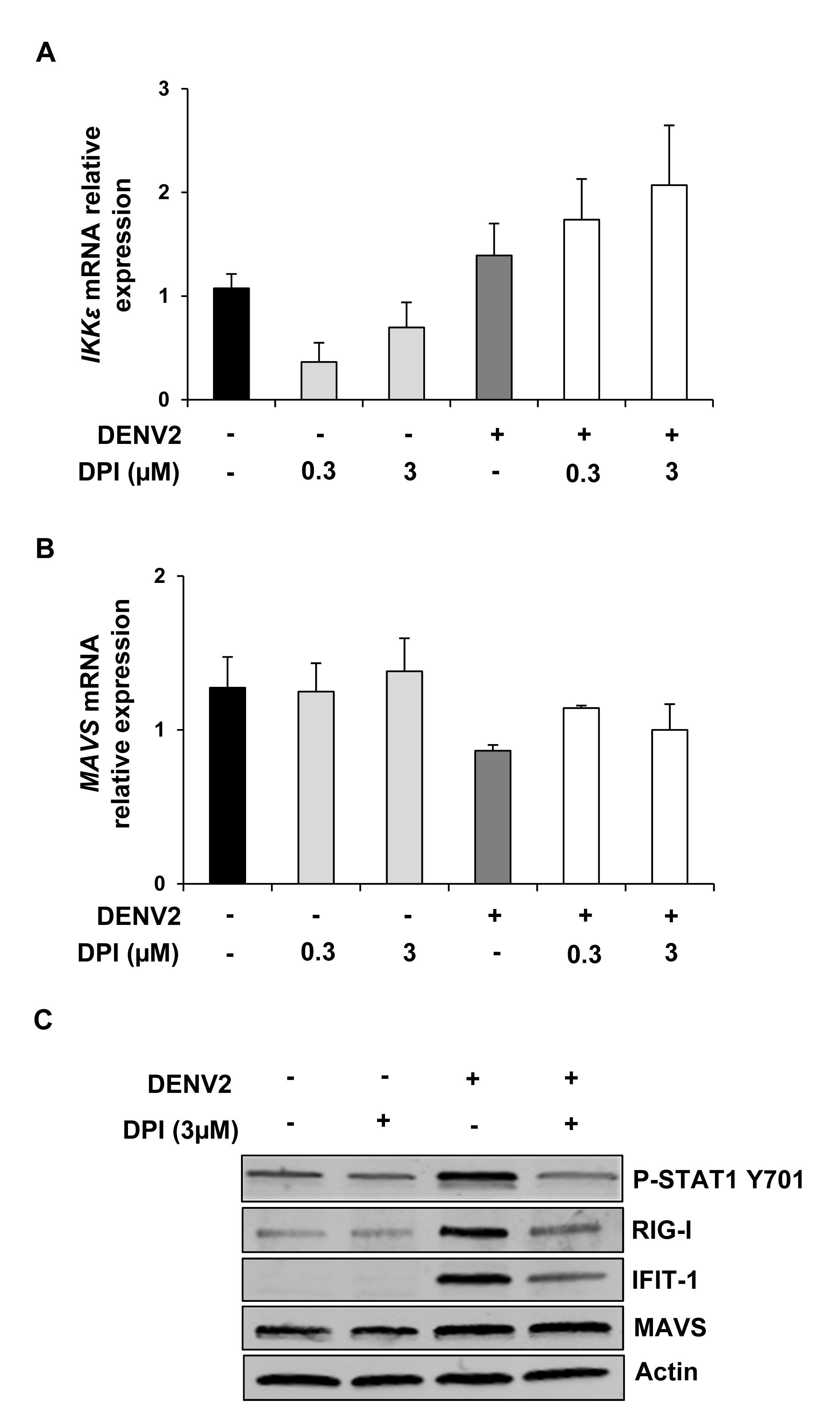

Supplement: S8 Figure — Chemical blocking of NOX affects neither MAVS nor IKKε expression. Mo-DC were pre-treated with the NOX inhibitor DPI (0.3–3 µM) for 1 h and subsequently infected with DENV2 (MOI 20). IKKε mRNA (A), MAVS mRNA (B) and MAVS protein (C) expression levels were detected by qPCR or immunoblotting, respectively. For panels (A) and (B), the data are the means ± SEM of one experiment performed in triplicate on three individual donors. For panel (C), the experiment has been performed on one donor. (TIF) [file ppat.1004566.s008.tif]
