# Supplementary material for: Cellular Oxidative Stress Response Controls the Antiviral and Apoptotic Programs in Dengue Virus-Infected Dendritic Cells
Source: PLoS Pathog. 2014 Dec 18;10(12):e1004566. doi: 10.1371/journal.ppat.1004566 (PMC4270780; doi:10.1371/journal.ppat.1004566)
Supplement: S1 Table — Primer sequences and probes used for the high throughput qPCR analysis. (DOCX) [file ppat.1004566.s009.docx]

| **TABLE S1** | | | | |
| --- | --- | --- | --- | --- |
| **Gene** |  | **Sequence (5' to 3')** | **Probe** | **Accession** |
| ACTB | F | attggcaatgagcggttc | 11 | NM_001101.3 |
|  | R | tgaaggtagtttcgtggatgc |  |  |
| ADAR | F | ttcgagaatcccaaacaagg | 39 | NM_001111.3 |
|  | R | ctggattccacagggattgt |  |  |
| APOBEC3A | F | gagaagggacaagcacatgg | 26 | NM_145699.3 |
|  | R | tggatccatcaagtgtctgg |  |  |
| B2M | F | ttctggcctggaggctatc | 42 | NM_004048.2 |
|  | R | tcaggaaatttgactttccattc |  |  |
| BIRC3 | F | gactgggcttgtccttgct | 44 | NM_001165.3 |
|  | R | aagaagtcgttttcctcctttgt |  |  |
| C1R | F | aacacgggccttgagaaat | 51 | NM_001733.4 |
|  | R | ttctgagggatgggaatgg |  |  |
| CASP1 | F | ccaggacattaaaataaggaaactgta | 4 | NM_033292.2 |
|  | R | ccaaaaacctttacagaaggatctc |  |  |
| CASP4 | F | ttgctttctgctcttcaacg | 80 | NM_001225.3 |
|  | R | gtgtgatgaagatagagccatt |  |  |
| CCL3 | F | tgctcagaatcatgcaggtc | 74 | NM_002983.2 |
|  | R | gcgtgtcagcagcaagtg |  |  |
| CCL3L1 | F | caggtcctctctgcacaact | 40 | NM_021006.4 |
|  | R | aatctgtcgggaggtgtagc |  |  |
| CXCL10 | F | gaaagcagttagcaaaggaaaggt | 34 | NM_001565.2 |
|  | R | gacatatactccatgtagggaagtga |  |  |
| DC-SIGN | F | cccagctcgtcgtaatcaa | 73 | AF290886.1 |
|  | R | ccaggtgaagcggttacttc |  |  |
| DDIT4 | F | ctggacagcagcaacagtg | 69 | NM_019058.2 |
|  | R | acaccccatccaggtaagc |  |  |
| DDX41 | F | agatggagaccatccgacac | 49 | NM_016222.2 |
|  | R | ctgcagcaaatccatgagg |  |  |
| DDX58 | F | tgtgggcaatgtcatcaaaa | 6 | NM_014314.3 |
|  | R | gaagcacttgctacctcttgc |  |  |
| DENV2 | F | atcctcctatggtacgcacaaa | 5 | gi_158976983 |
|  | R | ctccagtattattgaagctgctatcc |  |  |
| FCGR1 | F | gaccccatacagctggaaatc | 67 | NM_000566.3 |
|  | R | acctcaaggccagaggttct |  |  |
| FCGR2A | F | tgtgaccatcactgtccaagt | 5 | NM_001136219.1 |
|  | R | gctacagcagtcgcaatgac |  |  |
| GAPDH | F | agccacatcgctcagacac | 60 | NM_002046.3 |
|  | R | gcccaatacgaccaaatcc |  |  |
| GCLC | F | atgccatgggatttggaat | 80 | NM_001498.3 |
|  | R | gatcataaaggtatctggcctca |  |  |
| GCLM | F | gttggaacagctgtatcagtgg | 18 | NM_002061.2 |
|  | R | cagtcaaatctggtggcatc |  |  |
| HMOX-1 | F | ggcagagggtgatagaagagg | 15 | NM_002133.2 |
|  | R | agctcctgcaactcctcaaa |  |  |
| IDO1 | F | cagcgtctttcagtgctttg | 3 | NM_002164.4 |
|  | R | ggaggaactgagcagcatgt |  |  |
| IFI6 | F | aaccgtttactcgctgctgt | 40 | NM_002038.3 |
|  | R | gggctccgtcactagacctt |  |  |
| IFI16 | F | agagccatcttcggactcct | 42 | NM_005531.2 |
|  | R | tcattttggagattgtgtcttca |  |  |
| IFIH1 | F | ggcaccatgggaagtgatt | 20 | NM_022168.2 |
|  | R | gatgatgatattcttcccttcca |  |  |
| IFIT1 | F | gcctaatttacagcaaccatga | 50 | NM_001548.3 |
|  | R | tcatcaatggataactcccatgt |  |  |
| IFIT2 | F | atataggtctcttcagcatttattggt | 35 | NM_001547.4 |
|  | R | caaggaattcttattgttctcactca |  |  |
| IFITM1 | F | cacgcagaaaaccacacttc | 60 | NM_003641.3 |
|  | R | tgttcctccttgtgcatcttc |  |  |
| IFITM2 | F | tgaaccacattgtgcaaacc | 75 | NM_006435.2 |
|  | R | ctcctccttgagcatctcgt |  |  |
| IFITM3 | F | agatgctcaaggaggagcac | 76 | NM_021034.2 |
|  | R | gatgtggatcacggtggac |  |  |
| IFNA2 | F | aatggccttgacctttgctt | 49 | NM_000605.3 |
|  | R | cacagagcagcttgacttgc |  |  |
| IFNAR1 | F | atttacaccatttcgcaaagc | 65 | NM_000629.2 |
|  | R | cactattgccttatcttcagcttcta |  |  |
| IFNAR2 | F | tagcctccccaaagtcttga | 87 | NM_207585.1 |
|  | R | aaatgacctccaccatatcca |  |  |
| IFNB1 | F | ctttgctattttcagacaagattca | 20 | NM_002176.2 |
|  | R | gccaggaggttctcaacaat |  |  |
| IKKA | F | aggccatccactatgctgag | 65 | AF080157.1 |
|  | R | catgatttcagcatgcaaagac |  |  |
| IKKB | F | catccgatggcacaatca | 51 | 4185274 |
|  | R | ctggatctccaggcacca |  |  |
| IKKE | F | acctgtggcacacagatgac | 43 | AF241789.1 |
|  | R | accagctctccggatttctt |  |  |
| IL1A | F | tgacgccctcaatcaaagta | 66 | NM_000575.3 |
|  | R | tgacttataagcacccatgtcaa |  |  |
| IL1B | F | tacctgtcctgcgtgttgaa | 78 | NM_000576.2 |
|  | R | tctttgggtaatttttgggatct |  |  |
| IL6 | F | gatgagtacaaaagtcctgatcca | 40 | NM_000600.3 |
|  | R | ctgcagccactggttctgt |  |  |
| IL8 | F | agacagcagagcacacaagc | 72 | NM_000584.2 |
|  | R | tggttccttccggtggt |  |  |
| IL10 | F | gctggacaacttgttgttaaagg | 65 | NM_000572.2 |
|  | R | ctcagacaaggcttggcaac |  |  |
| IL28A | F | ccagttccgggcctgtat | 79 | NM_172138.1 |
|  | R | gccaggggactccttttt |  |  |
| IL28RA | F | cccccactggatctgaagta | 12 | NM_170743.2 |
|  | R | gagtgactggaaatagggtcttg |  |  |
|  |  |  |  |  |
| IL29 | F | ggcctgtatccagcctca | 79 | NM_172140.1 |
|  | R | aaggtgacagatgcctccag |  |  |
| IRF1 | F | gggctgtcagttgattctgg | 57 | NM_002198.2 |
|  | R | ctatggcacatgcctcaaaa |  |  |
| IRF3 | F | cttggaagcacggcctac | 18 | NM_001571.4 |
|  | R | cgggaacatatgcaccagt |  |  |
| IFR7 | F | agctgtgctggcgagaag | 72 | NM_001572.3 |
|  | R | ttggagtccagcatgtgtg |  |  |
| IRF9 | F | atgcaggcaagcaggactt | 89 | NM_006084.4 |
|  | R | tcctgtgtccccctccttat |  |  |
| ISG15 | F | gcgaactcatctttgccagta | 23 | NM_005101.3 |
|  | R | ccagcatcttcaccgtcag |  |  |
| KEAP1 | F | accacaacagtgtggagaggt |  | NM_203500.1 |
|  | R | cgatccttcgtgtcagcat |  |  |
| MAP3K14 | F | ccagctgccatctctatcatc | 64 | NM_003954.2 |
|  | R | aaaaggtggggctgaactct |  |  |
| MAVS | F | tgcagcaatggtatctgcat | 39 | NM_020746.3 |
|  | R | aaatgattcagcgggagaaa |  |  |
| MRC1 | F | caccatcgaggaattggact | 14 | NM_002438.2 |
|  | R | acaattcgtcatttggctca |  |  |
| MX1 | F | ttcagcacctgatggccta | 79 | NM_001144925.1 |
|  | R | aaagggatgtggctggagat |  |  |
| MX2 | F | cagacctgaccatcattgacc | 9 | NM_002463.1 |
|  | R | tgatgagagccttgatctgc |  |  |
| NFE2L2 | F | ccggcatttcactaaacaca | 9 | NM_006164.3 |
|  | R | tgtgtctccatagctggaagatt |  |  |
| NFKB1 | F | accctgaccttgcctatttg | 39 | NM_003998.2 |
|  | R | agctctttttcccgatctcc |  |  |
| NQO1 | F | acctctgcctcccaggtt | 2 | NM_000903.2 |
|  | R | cgcctgtcatcccagct |  |  |
| OAS3 | F | gacggatgttagcctgctg | 43 | NM_006187.2 |
|  | R | tggggatttggtttggtg |  |  |
| OASL | F | ttgctatgacaacagggagaac | 78 | NM_003733.2 |
|  | R | ctgtcaagtggatgtctcgtg |  |  |
| PMAIP1 | F | ggagatgcctgggaagaag | 11 | NM_021127.2 |
|  | R | ccaaatctcctgagttgagtagc |  |  |
| RELA | F | tcatgaagaagagtcctttcagc | 39 | NM_021975.3 |
|  | R | ctggcttggggacagaag |  |  |
| RIPK1 | F | gtgtacaaggggcccaact | 25 | NM_003804.3 |
|  | R | cggctgtgtctcagtctgtt |  |  |
| RSAD2 | F | tgcttttgcttaaggaagctg | 39 | NM_080657.4 |
|  | R | aggtattctccccggtcttg |  |  |
| SERPING1 | F | catcgccagcctccttac | 15 | NM_000062.2 |
|  | R | gaggatgctctccaggtttg |  |  |
| SOCS1 | F | cccctggttgttgtagcag | 36 | NM_003745.1 |
|  | R | gtaggaggtgcgagttcagg |  |  |
| SOCS3 | F | gacctgaagggaaccatcct | 55 | NM_003955.3 |
|  | R | tgtgttttcggtgactgtcc |  |  |
| SOD2 | F | gcactagcagcatgttgagc | 1 | NM_000636.2 |
|  | R | ccgtagtcgtagggcaggt |  |  |
| SQSTM1 | F | agctgccttgtacccacatc | 14 | NM_003900.4 |
|  | R | cagagaagcccatggacag |  |  |
| STAT1 | F | ggatcagctgcagaactggt | 74 | NM_007315.3 |
|  | R | tttctgttccaattcctccaa |  |  |
| TANK | F | gaggaatagtctacaaaggaagacttg | 80 | NM_004180.2 |
|  | R | actataaaggatggagtaaatgacagg |  |  |
| TAP1 | F | gcaagaaataaagacactcaacca | 75 | NM_000593.5 |
|  | R | cccactttcagcagcatacc |  |  |
| TICAM1 | F | catgccatggttctcctga | 72 | NM_182919.2 |
|  | R | cggccttcaatgcctcta |  |  |
| TLR3 | F | aaggctagcagtcatccaaca | 22 | NM_003265.2 |
|  | R | agcaacttcatggctaacagtg |  |  |
| TLR7 | F | ccagtgtctaaagaacctggaaac | 5 | NM_016562.3 |
|  | R | tcagggacagtggtcagttg |  |  |
| TLR8 | F | tgtggttgttttctggattcaa | 79 | NM_138636.4 |
|  | R | gctcgcatggcttacatga |  |  |
| TNF | F | cagcctcttctccttcctgat | 40 | NM_000594.2 |
|  | R | gccagagggctgattagaga |  |  |
| TNFAIP6 | F | ggccatctcgcaacttaca | 34 | NM_007115.2 |
|  | R | gcagcacagacatgaaatcc |  |  |
| TNFSF10 | F | tcacagtgctcctgcagtct | 5 | NM_003810.2 |
|  | R | tggagtacttgtcctgcatctg |  |  |
| TRAF3 | F | caagtggctcggaacacag | 33 | NM_145725.1 |
|  | R | cactcagcatctggtcatgc |  |  |
| TRAF6 | F | ccaagcagcagtgaccatag | 2 | NM_145803.2 |
|  | R | acatccccattccacacagt |  |  |
| TXNDR1 | F | tgaggagaaagctgtggagaa | 60 | NM_003330.2 |
|  | R | ccattccaatggccaaaa |  |  |
| XAF1 | F | cctgccgatcctaaatcaac | 2 | NM_017523.2 |
|  | R | tttccttttgatgaagctaacca |  |  |
| XBP1 | F | ggagttaagacagcgcttgg | 37 | NM_005080.3 |
|  | R | cactggcctcacttcattcc |  |  |
